# Solution Ensembles of Griselimycin Expand the Definition of Molecular Chameleonicity

**DOI:** 10.1021/acs.jmedchem.6c01558

**Published:** 2026-07-21

**Authors:** Luca J. Hagemeyer, William T. P. Darling, Nele-Johanna Hempel-Højgaard, Jan Kihlberg, Mate Erdelyi

**Affiliations:** † Department of Chemistry for Life Sciences, 8097 Uppsala University, Uppsala SE 751 23, Sweden; ‡ Novo Nordisk A/S, Therapeutics Discovery, Research & Development, Novo Nordisk Park 1, 2760 Måløv, Denmark; § Center of Excellence for the Chemical Mechanisms of Life, Uppsala University, Uppsala SE 751 23, Sweden

## Abstract

Achieving satisfactory permeability for beyond the rule of 5 (bRo5) molecules, such as bioactive macrocycles, remains a challenge for drug discovery. Gaining a mechanistic understanding of the correlation between their structure and permeability is expected to improve the foundation for rational prediction of the pharmacokinetic behavior of novel macrocyclic drug candidates. Based on a solution-phase NMR investigation, we suggest that griselimycin, a macrocyclic peptide that has remarkable antibiotic activity, permeates membranes while exposing its polar moieties in an apolar membrane-like environment and hiding them in a polar environment. This noncanonical behavior is driven by the simultaneous exposure of lipophilic moieties in an apolar environment and by their hydrophobic collapse in aqueous solution. The unexpected behavior of griselimycin opposes the concept of classical chameleonicity and may be described as “inverse chameleonicity”.

## Graphical abstract

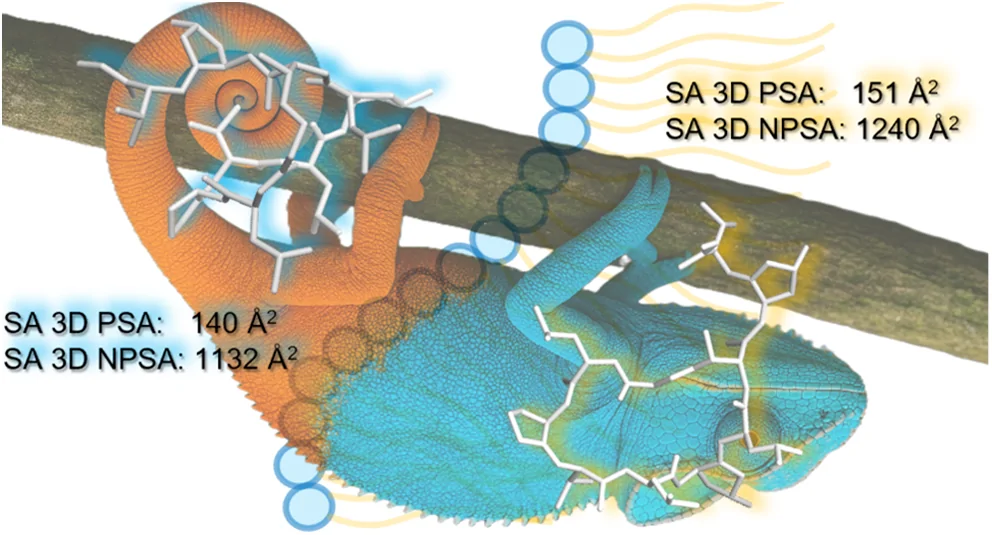

## Introduction

Over the past few decades, macrocyclic compounds have garnered increasing attention in drug discovery. They are particularly interesting due to their ability to combine high specificity for difficult-to-drug targets^1–3^ with notable cell membrane permeability and oral bioavailability despite their size.^1,4^ This dual characteristic is highly sought after when developing novel therapeutics.

Among macrocycles, macrocyclic peptides have come into fashion in recent decades.^5,6^ The conformational restriction imposed by macrocyclization confers increased resistance against proteases, as compared to linear peptides. Even more importantly, DNA and RNA display technologies have made it possible to produce and screen vast libraries consisting of up to 10–100 billion peptidic macrocycles, potentially allowing one to find ligands for any target of interest. It is now also possible to incorporate *N*-methylated and structurally diverse non-natural amino acids into such libraries.^7^ LUNA18 (paluratide), a recently developed, orally bioavailable 11-mer cyclic peptide inhibitor of the intracellular target KRAS, is a notable example of the opportunities provided by these technologies.^7,8^ Another illustrative example is the successful de novo design of head-to-tail cyclized, cell-permeable macrocyclic peptides that inhibit the Cyclin A/B RxL interaction and show antitumor activity.^9,10^

Since the turn of the millennium, drug discovery has been guided by Lipinski’s Rule of Five (Ro5),^11^ which sets four physicochemical property constraints outside of which poor cell permeability, solubility, and oral absorption are more likely to be encountered. Drug discovery has lately gradually moved beyond these constraints to explore the beyond Rule of Five (bRo5) chemical space.^14–16^ Besides macrocycles, new modalities such as Proteolysis Targeting Chimeras (PROTACs),^12,13^ oligonucleotides, and drug conjugates populate the less explored bRo5 space.^17^ The pharmacokinetics of bRo5 compounds remains poorly understood; for example, some exhibit unexpected membrane permeability, while others with a similar structure do not. The extent to which compounds are capable of acting as molecular chameleons may be the key to understanding the subtle structural differences responsible for the differences in permeabilities.^18,19^ This highlights the need for deeper investigation into the structural features governing passive, transcellular cell permeability in the bRo5 space; such knowledge is expected to contribute to improved design of orally bioavailable drugs. It should, however, be kept in mind that passive cell permeability is one of several compound properties that are required for oral bioavailability. Others include the solubility in the gastrointestinal tract, affinity for transporters involved in efflux or, more rarely, in uptake across intestinal epithelial cells, as well as metabolism in the liver and in the intestine. In addition, the oral bioavailability may depend on the dose and formulation used for administration to the patient.

Griselimycin (Figure 1) is a naturally occurring antibiotic that has been neglected for decades, yet recently received increased attention due to the rapid spread of microbial resistance.^20–22^ Griselimycin exhibits strong selectivity for the β-subunit of DNA polymerase in Gram-negative pathogens^22^ as well as in *Mycobacterium tuberculosis* (Figure 1).^21^ These attributes make it an excellent candidate for further study, particularly in the context of combating multidrug-resistant (MDR) bacterial strains.^21^ However, its applicability as a drug is hampered by its poor pharmacokinetic profile. While its oral bioavailability is excellent for a molecule of this size, its short plasma elimination half-life (*t*_1/2_) necessitates the development of structural alternatives.^21^

**Figure 1:**
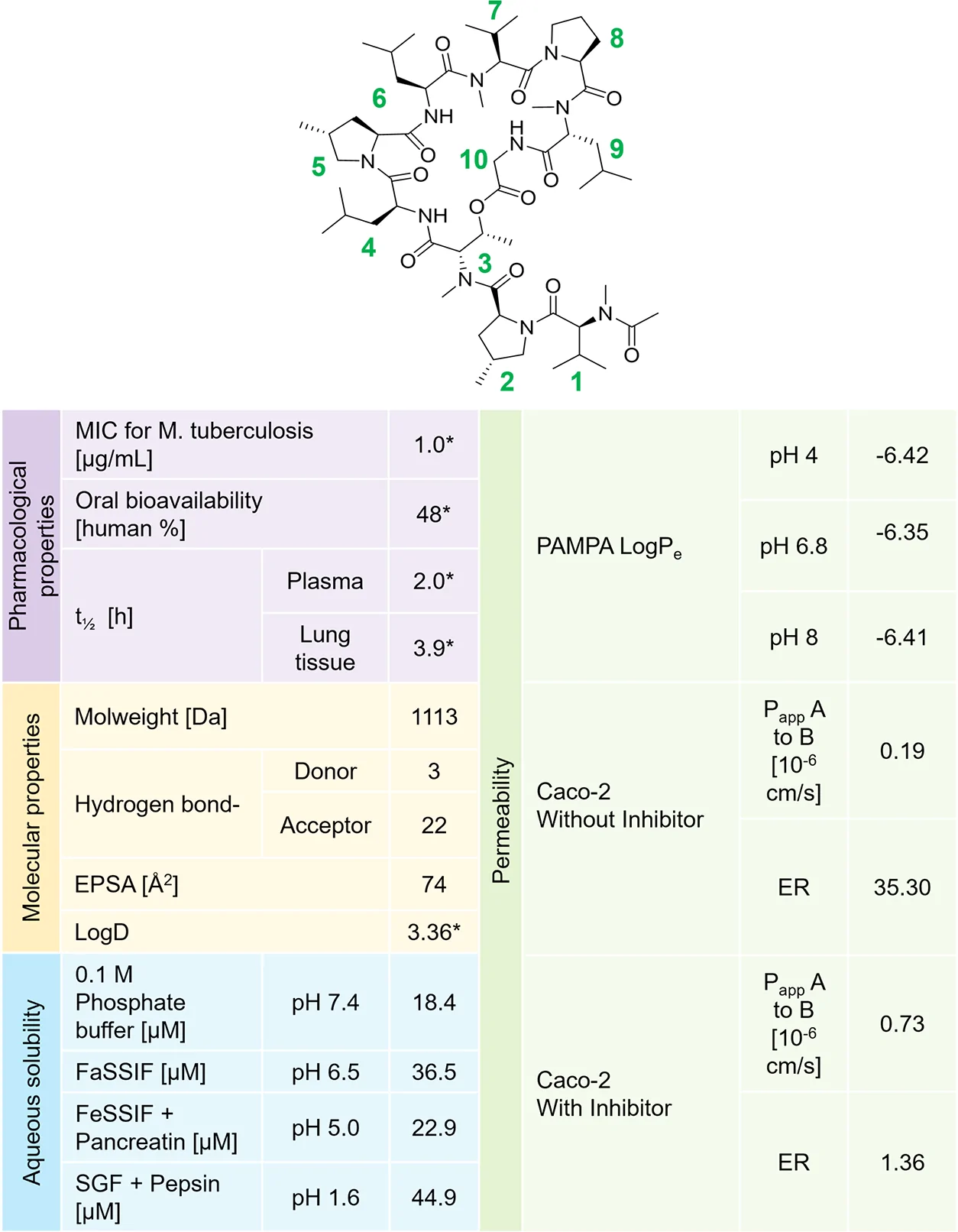
Structure, physicochemical, and pharmacological properties of griselimycin. *Data reported previously.^21,24^ Elacridar was used to inhibit efflux by P-glycoprotein in the Caco-2 assay. Here, EPSA is the experimental polar surface area, ER is the efflux ratio, FaSSIF is the fasted-state simulated intestinal fluid, FeSSIF is the fed-state simulated intestinal fluid, and SGF is the simulated gastric fluid.

The solution ensemble of the octapeptide core of methyl-griselimycin in an apolar environment has been determined using NMR spectroscopy.^23^ Herein, we provide a comprehensive analysis of the conformational behavior of griselimycin, including its dipeptide tail, in both polar and apolar environments, along with its polarity, aqueous solubility, and membrane permeability. Interpretation of these results provides novel mechanistic insights into the permeability and solubility of macrocyclic peptides, expectably promoting the design of future macrocyclic peptide drugs.

## Results and Discussion

### Description and Characterization

Griselimycin features a dipeptide tail attached to an octapeptide macrocyclic core, which is cyclized via the formation of a lactone to the hydroxyl group of Thr3 (Figure 1). It contains 4-methylproline and d-leucine, two unusual amino acids. The highly lipophilic nature of griselimycin (LogD 3.36)^24^ originates from its hydrophobic amino acids and from the high degree of *N*-methylation of its amide backbone. Additionally, two of its three proline residues are methylated at the γ-position, and its N-terminus is acetylated.

We determined the polarity, aqueous solubility, and membrane permeability of griselimycin, data relevant for its pharmacokinetic behavior (Figure 1). The experimental polar surface area (EPSA) of griselimycin is 74 Å^2^, well below the 90 Å^2^ that has been reported as the limit below which macrocyclic peptides are expected to be cell membrane permeable.^25,26^ Accordingly, griselimycin was permeable both in the parallel artificial membrane permeability assay (PAMPA) and across Caco-2 cell monolayers; however, permeability was relatively low (Figure 1). The Log *P*_e_ of griselimycin is below −6, which is the reported cutoff for low passive permeability in the PAMPA assay. As expected from the lack of charged groups, its permeability was pH independent (Figure 1, Table S33). Both in the absence and presence of efflux transporter inhibitors, the permeability across Caco-2 cells remained low (*P*_app_ A → B < 1 × 10^–6^ cm/s) (Figure 1, Table S34, Supporting Information). The efflux ratio (ER 35) indicated griselimycin to be highly susceptible to efflux transporters. Addition of elacridar in the Caco-2 assay inhibited efflux, revealing it to be caused mainly by P-glycoprotein.

Phosphate buffer, fasted-state simulated intestinal fluid (FaSSIF), fed-state simulated intestinal fluid (FeSSIF) with pancreatin, as well as simulated gastric fluid (SGF) with pepsin, were used to determine the solubility of griselimycin in media that mimic the different compartments and pH in the digestive system and the bloodstream (Figure 1). The aqueous solubility of griselimycin in these biorelevant media was low and did not show any major differences between the media (Figure 1). This is in line with expectations, as griselimycin has a high lipophilicity and remains uncharged across the physiological pH range of the solubility assays.

### Protocol for the Determination of Conformational Ensembles

To gain an understanding of a possible conformational change associated with the membrane permeation of griselimycin, we determined its solution ensembles in environments resembling the extracellular space and the cell membrane. As model solvents, we chose CDCl_3_ (ε = 4.8) since its polarity resembles that of the interior of the cell membrane (ε = 3.0),^27^ and D_2_O:DMSO-*d*_6_ (4:1) because its polarity (ε ≈ 73)^28^ is similar to that of the extra- and intracellular environments (ε ≈ 80) and as griselimycin’s low aqueous solubility prohibits NMR studies inerugi water (Figure 1). These solvents obviously do not mimic the extracellular and the membrane environments in every aspect; however, the polarity difference between D_2_O:DMSO-*d*_6_ (4:1) and CDCl_3_ is a reasonable model for the polarity change a compound experiences when diffusing from the extracellular space into the cell membrane. This approximation has commonly been used when addressing the membrane permeability of drug candidates.^29–32^ We identified conformational ensembles using the NAMFIS algorithm,^33,34^ which has previously been successfully applied to describe the conformational ensembles of macrocycles^32,35,36^ as well as of other flexible drug candidates.^30,31,37^ In short, NAMFIS deconvolutes population-averaged NOE-based interproton distances and ^3^ *J*_HH_ scalar coupling constant-based dihedral angles (Figure 2) to identify the conformers that are present in solution, along with their molar fractions. The algorithm selects conformers from a theoretically feasible conformer pool by nonlinear least-squares fitting of the back-calculated population-weighted NMR parameters of each theoretical conformer to those experimentally observed. We derived interproton distances from NOESY (CDCl_3_) or EASY-ROESY^38^ (D_2_O:DMSO-*d*_6_ (4:1)) build-ups, acquired with seven mixing times (for details see Methods and Table S5, Supporting Information) and ^3^ *J*_HH_s from ^1^H NMR spectra in H_2_O:DMSO-*d*_6_ (4:1). EASY-ROESY was used for the D_2_O:DMSO-*d*_6_ (4:1) solution due to the slower tumbling time in a more viscous solvent mixture. A theoretical conformational pool covering the entire conformational space available for griselimycin was produced by combining the outputs of eight independent Monte Carlo Multiple Minima (MCMM) conformational searches. These used four different force fields (see Methods for details), each with implicit water and chloroform solvent models (Tables S9–S12, Supporting Information) and a 42 kJ/mol energy cutoff. Redundant conformers were eliminated using a 0.9 Å heavy atom RMSD cutoff. All amide bonds were kept *trans* configured,^39,40^ except that of Pro-8, which was restrained into *cis*-amide configuration based on the ^13^C chemical shifts of its C_γ_ and C_β_,^41^ and on the Val-7-H_α_ and Pro-8-H_α_ NOE observed both for CDCl_3_ and D_2_O:DMSO-*d*_6_ (4:1) solutions. Pro-8 possessing a *cis*-amide bond was in line with the independent observations made for methyl-griselimycin, a close structural analogue.^22,23^ To avoid contamination of the data with errors due to the limited ability of the theoretical conformational search in simultaneously describing the motion of every side chain, the conformational ensemble of the macrocyclic ring was solved first using NOEs and *J*s of the backbone protons. A subsequent conformational search was performed for each macrocycle conformer of the ensemble, exploring the orientations of Val-1 and MePro-2 while keeping the backbone geometries fixed. The output ensembles were validated by Jackknife resampling,^42,43^ and by the introduction of ±5% random noise on the experimental data (for details, see Methods and Tables S15, S20, and S21, Supporting Information). Conformational families were defined based on the backbone RMSD (Tables S25–S27, Supporting Information).^32,33^

**Figure 2:**
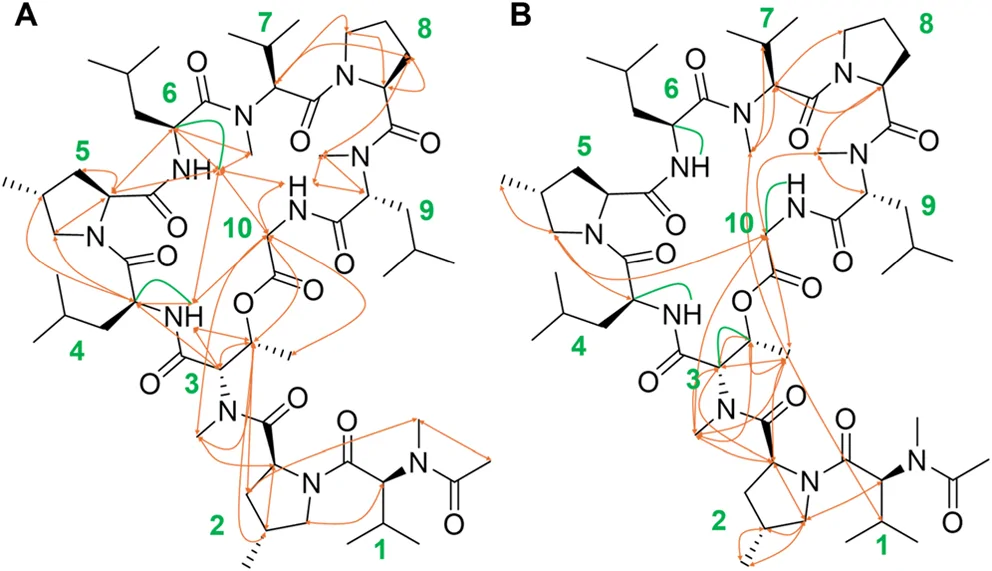
NOEs/ROEs (orange) and scalar couplings (green) used in the NAMFIS analysis of griselimycin, observed (A) for CDCl_3_ and (B) for D_2_O:DMSO-*d*_6_ (4:1) and H_2_O:DMSO-*d*_6_ (4:1) solutions at 25 °C. For details, see Tables S3–S7 in the Supporting Information. Amino acid numbering is given in green.

### Conformational Ensemble in CDCl_3_

Griselimycin adopts one dominant conformation (88%, family A) in chloroform, with four distinct subfamilies (Figure 3 and Table S18, Supporting Information). The macrocyclic core is flat and elongated, with residues Val-1 and MePro-2 being bent out of the macrocycle’s plane for subfamilies A1 and A2 (66%) while extended for subfamilies A3 and A4 (21%). Almost all apolar amino-acid side chains are solvent exposed, whereas the hydrogen bond donors and acceptors of the macrocycles are oriented inward. The low amide temperature coefficients (Δδ/Δ*T*) of Leu-4, Leu-6, and Gly-10 reveal these amide protons to be intramolecularly hydrogen bonded (Table S8, Supporting Information).^44^ Importantly, the observed chemical shift, similar to the NOE and *J*, is the population-weighted average of those of existing conformers. Changing the temperature within a 25 °C interval slightly alters the molar fraction of the existing solution conformers; however, the formation of any new conformer is not expected. Accordingly, Δδ/Δ*T* provides helpful complementary information to NOEs and *J*s, reporting on the possible involvement of amide protons in intramolecular hydrogen bonding. Subfamily A1, making 41% of the solution ensemble, features an oval macrocyclic core and its dipeptide tail bent out of the plane of the macrocycle as well as two intramolecular hydrogen bonds between NH_Leu‑6_ – CO_Leu‑4_ and NH_Gly‑10_ – CO_Leu‑6_ (Figure 3, Table S19, Supporting Information). This conformer shows high similarity (heavy atom RMSD 1.82 Å) to griselimycin bound to the β-subunit of DNA polymerase III from various Gram-negative bacteria, observed by single-crystal X-ray diffraction (Figure 4).^22^ Conformational subfamily A4 also resembles the target-bound conformation (heavy atom RMSD 1.39 Å),^22^ but has a different pattern of intramolecular hydrogen bonds. Subfamily A2, which makes 25% of the solution ensemble, displays a slight kink between the macrocyclic core and its “tail” (Figure 3). Two of the three amide protons form intramolecular hydrogen bonds, and HN_Leu‑6_ is in close proximity to the carbonyl of Leu-4, potentially allowing the formation of an additional intramolecular hydrogen bond (Figure 3, Table S19, Supporting Information). Subfamily A3, with 13%, is defined by a narrower oval-shaped core with NH_Leu‑6_ and NH_Gly‑10_ forming hydrogen bonds to CO_Leu‑4_, whereas NH_Leu‑4_, which is rotated, may form a hydrogen bond to CO_MePro‑2_. Conformational family B (12%) lacks intramolecular hydrogen bonds and is noticeably more bent, most similar to the conformations observed in D_2_O:DMSO-*d*_6_ (4:1) solution.

**Figure 3:**
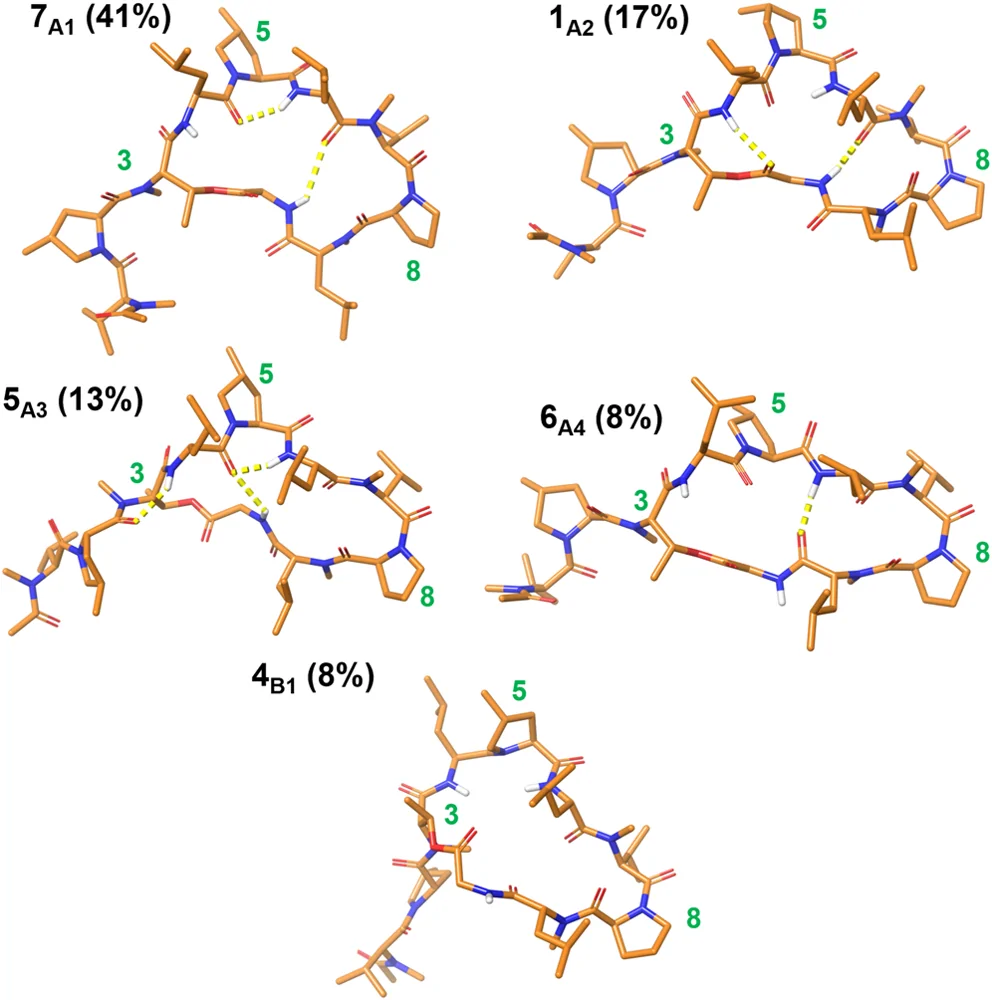
Major conformations of griselimycin from each subfamily of family A and B in CDCl_3_. Conformation numbers, with conformation family in subscript and population in parentheses, are given in black; amino acid numbering is given in green.

**Figure 4:**
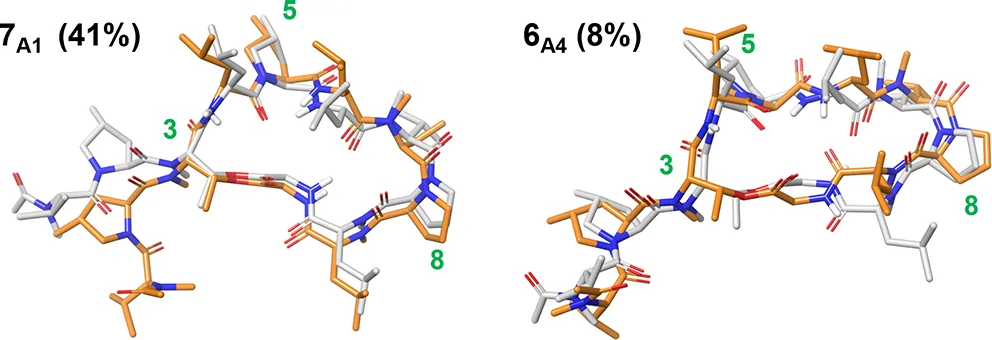
A1 and A4 conformations (orange) superimposed with the griselimycin geometry when bound to the β-subunit of DNA polymerase III of *Bartonella birtlesii* (PDB 6PTR)^22^ (gray). Conformation numbers, with conformation family in subscript and population in parentheses, are given in black; amino acid numbering is given in green.

### Conformational Ensemble in D_2_O:DMSO-*d*_6_ (4:1)

Griselimycin shows higher conformational diversity in a polar solvent mixture (Figure 5), with three distinct conformational families. Family A, 50% of the solution ensemble, possesses a characteristic saddle-shaped conformation with the Val-1 and MePro-2 “tail” being bent over the macrocycle (Figure 5, Table S30, Supporting Information). While conformation A1 forms one intramolecular hydrogen bond between NH_Leu‑6_ and CO_Leu‑4_, subfamily A2 shows a β sheet-like structure^45,46^ with intramolecular hydrogen bonds between NH_Leu‑6_ – CO_Gly‑10_ and NH_Gly‑10_ – CO_Leu‑6_ (Table S30, Supporting Information). Family B, with 38%, has a flatter, more open macrocycle conformation, an altering orientation of the Val-1 and MePro-2 “tail” (B1 and B2), and only subfamily B1 shows an intramolecular hydrogen bond between NH_Leu‑6_ and CO_Thr‑3_. Conformational family C (11%) has an extended macrocyclic core with the apolar side chains being oriented toward each other.

**Figure 5:**
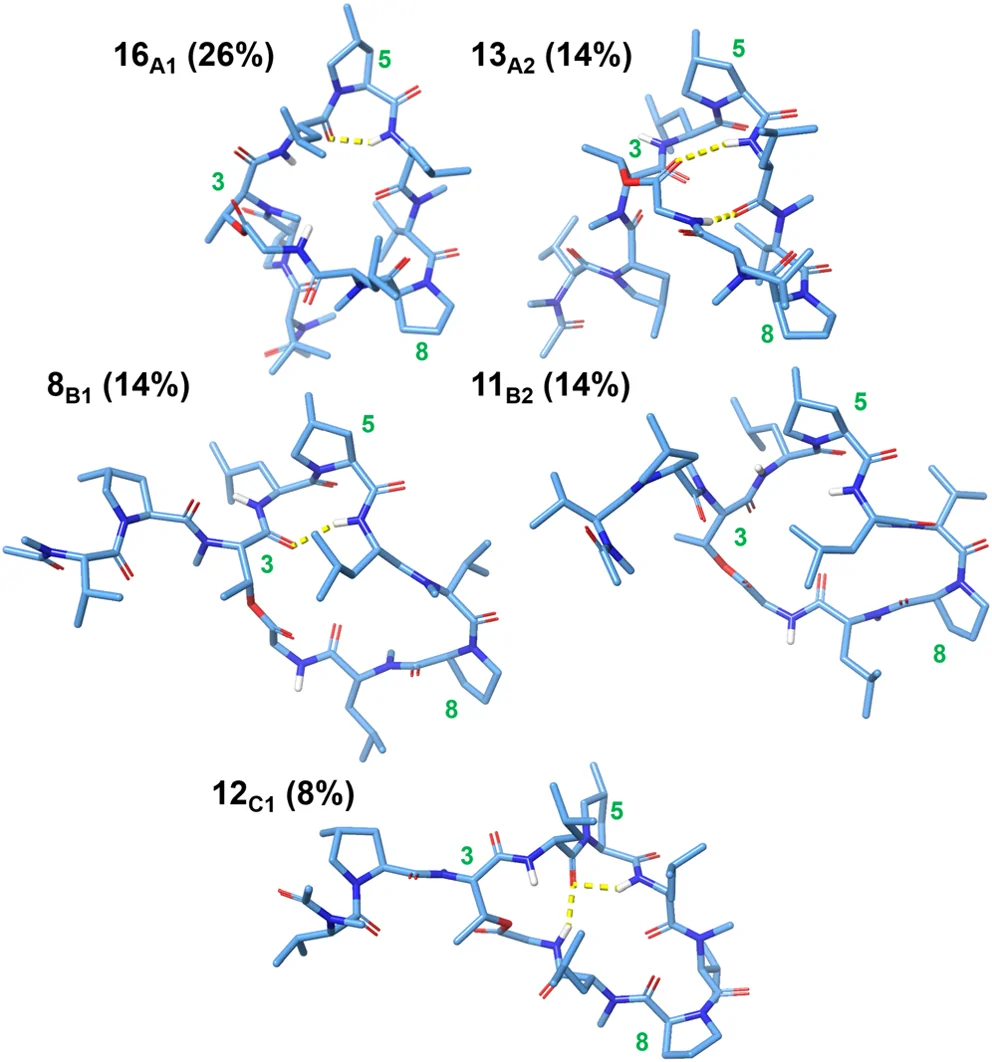
Griselimycin conformations in D_2_O:DMSO-*d*_6_ (4:1). Conformation numbers, with conformation family in subscript and population in parentheses, are given in black; amino acid numbering is given in green.

### Characteristics of Conformational Ensembles

The passive permeability of a compound across a cell membrane is determined by its size and polarity.^47^ For compounds in the bRo5 space, the radius of gyration (*R*_gyr_),^48^ solvent-accessible 3D polar surface area (SA 3D PSA),^49^ and solvent-accessible 3D nonpolar surface area (SA 3D NPSA)^29^ are descriptors of size, polarity, and lipophilicity reported to correlate better to permeability than MW, TPSA, and * c* log *P*. In addition to visual assessment, we therefore characterized the solution ensembles of griselimycin using these three descriptors.

In D_2_O:DMSO-*d*_6_ (4:1), the conformations have a lower *R*_gyr_, SA 3D PSA, and SA 3D NPSA than those in the membrane-mimicking CDCl_3_ ensemble (Figure 6, Tables S31–32, Supporting Information). At first glance, the increase in polarity, i.e., in SA 3D PSA, when griselimycin transitions from an aqueous into an apolar, membrane-mimicking environment may appear surprising. However, this conformational behavior most likely arises from the environment-dependent orientations of the apolar amino-acid side chains, in synergy with the apolar nature of the *N*-methylated amide backbone. In D_2_O:DMSO-*d*_6_, the apolar residues undergo a hydrophobic collapse toward the molecular core, which also buries the polar moieties. In chloroform, the apolar side chains are exposed to the solvent, necessitating the simultaneous exposure of the polar moieties.

**Figure 6:**
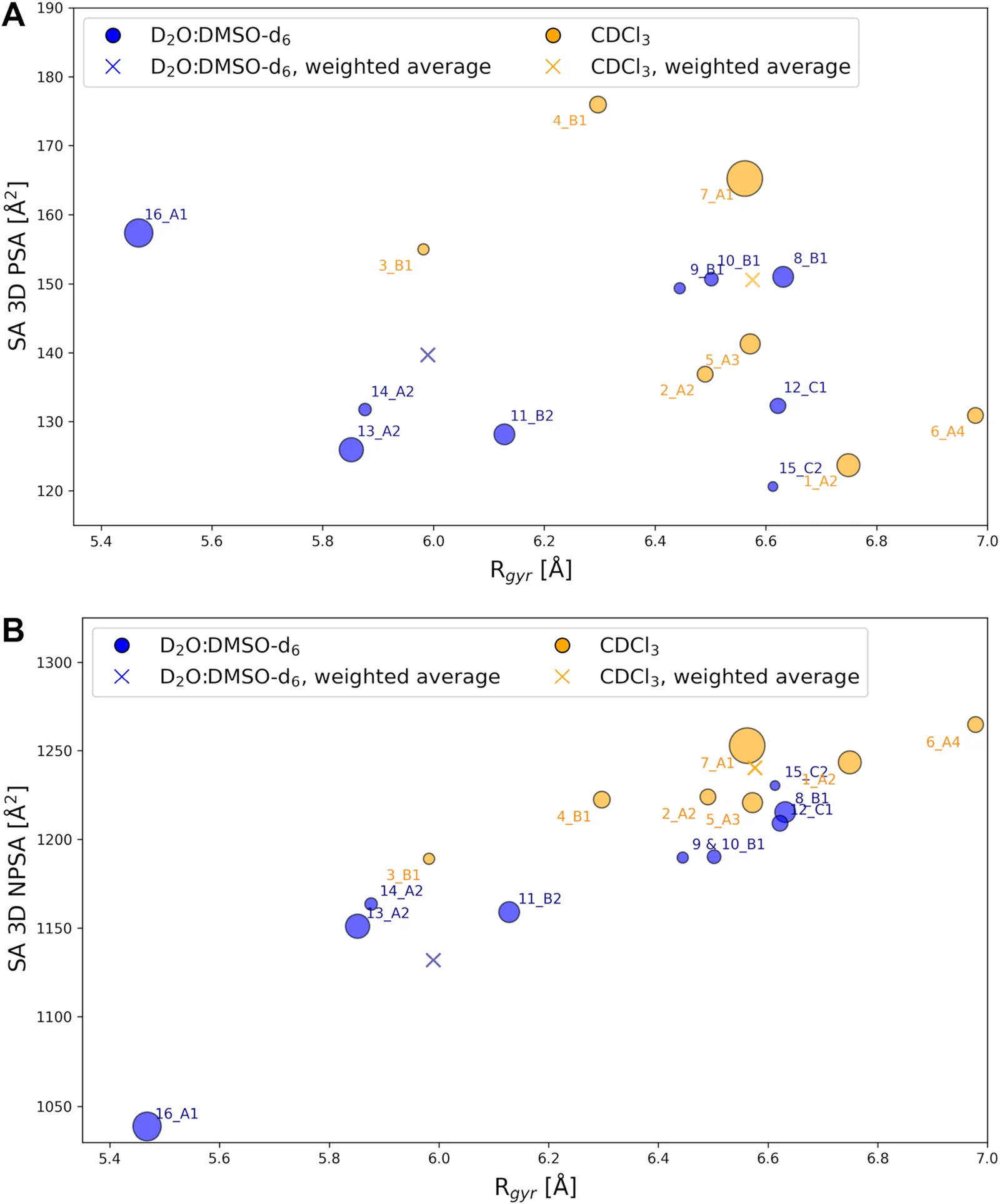
(A) Solvent-accessible 3D polar surface area (SA 3D PSA) versus radius of gyration (*R*_gyr_) and (B) solvent-accessible 3D nonpolar surface area (SA 3D NPSA) versus radius of gyration (*R*_gyr_) for the solution conformations of griselimycin in CDCl_3_ (orange) and in D_2_O:DMSO-*d*_6_ (4:1) (blue). The size of the circle for a conformation is proportional to its population (in %), while the number identifies the conformations and the letter code after the underscore identifies the family and subfamily. The X designates the population-weighted average of the descriptors for the ensemble indicated by its color.

### Comparison of Griselimycin and Cyclosporine A

Griselimycin and cyclosporine A (Figure 7) are both macrocyclic peptides located far into the beyond rule of five space (MWs 1100–1200 Da). With the exception of the first residue of cyclosporine A, both are entirely composed of amino acids exposing apolar side chains and are extensively *N*-methylated. Cyclosporine A is a head-to-tail cyclized undecapeptide, while griselimycin is a proline-rich, head-to-side chain cyclized decadepsipeptide consisting of an octapeptide core linked to a dipeptide tail.

**Figure 7:**
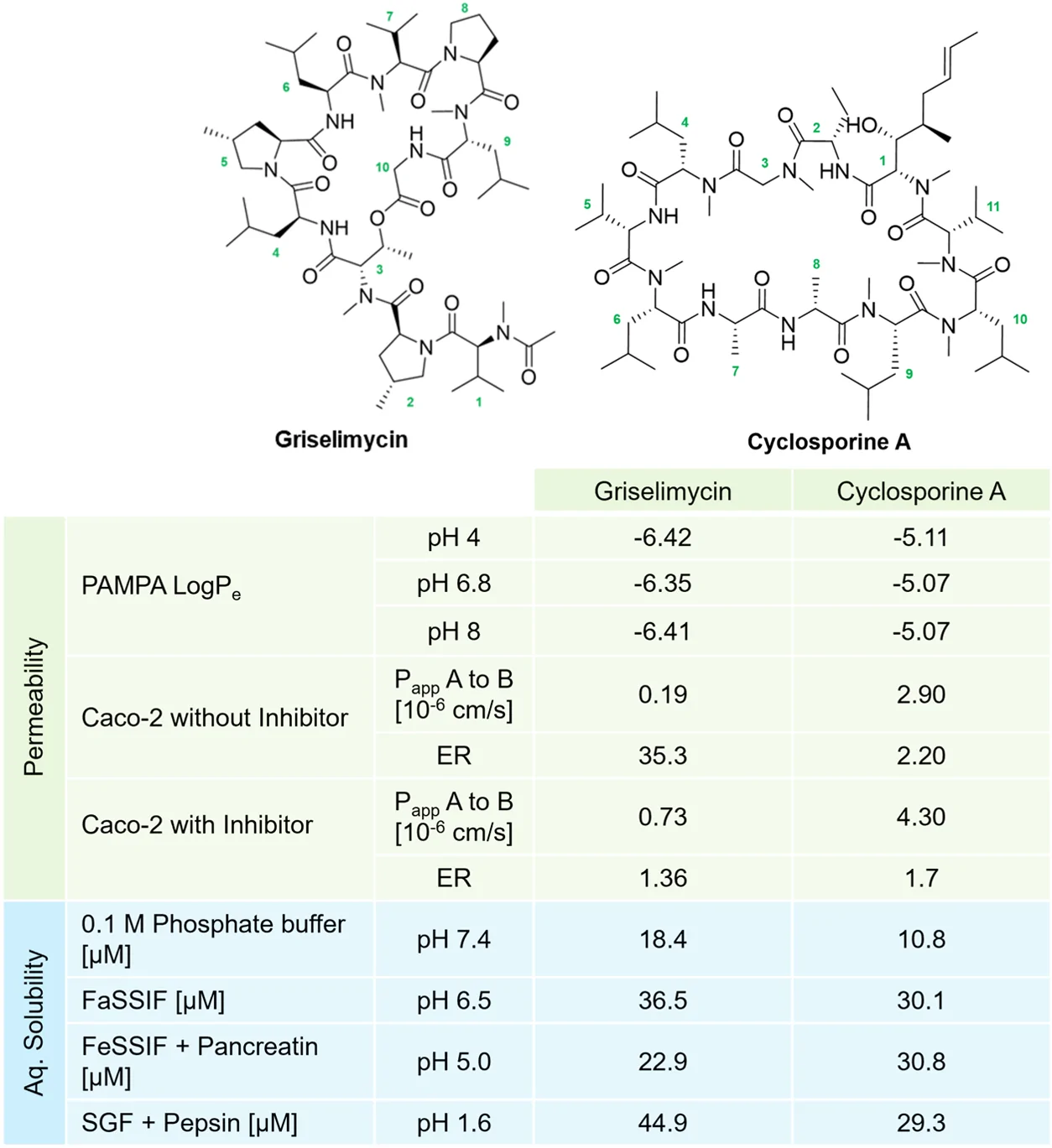
Comparison of the experimental properties of griselimycin and cyclosporin A. Elacridar was used to inhibit efflux by P-glycoprotein in the Caco-2 assay. Here, ER is the efflux ratio, FaSSIF is the fasted-state simulated intestinal fluid, FeSSIF is the fed-state simulated intestinal fluid, and SGF is the simulated gastric fluid.

Despite their overall structural similarities, griselimycin and cyclosporine A exhibit substantially different permeabilities (Figure 7). Hence, cyclosporine A has a 20-fold higher passive membrane permeability in the PAMPA assay and a 6-fold higher permeability across efflux-inhibited Caco-2 cell monolayers. In the absence of any inhibitors, cyclosporine A also proves to be much less susceptible to efflux transporters than griselimycin. Overall, the membrane permeability of cyclosporin A thus falls in the moderate range, while that of griselimycin is low. The solubilities of the two cyclic peptides in biorelevant media are comparable (≤1.7-fold difference in each of the four media), but above 10 μM, which is often considered as a lower limit for acceptable solubility in drug development.

The moderate cell permeability of cyclosporine A has been attributed to its ability to behave as a molecular chameleon,^18^ i.e., that it adopts conformations having low polarity when crossing a cell membrane and more polar ones in aqueous environments. A predominant (95%) DEKSAN-type conformation (CSD code DEKSAN, Figure 8), also identified by NMR spectroscopy in chloroform,^50,51^ is considered essential for permeability.^20,48^ Computational and NMR studies have proposed additional, less populated conformations, in exchange with the DEKSAN-type conformation, to be of importance for permeability.^52–54^ The dominant DEKSAN-type conformation is characterized by an extensive network of intramolecular hydrogen bonds (IMHBs) that effectively shield all four amide protons of the macrocyclic backbone from the surrounding environment. Formation of this “closed” conformation depends on the adoption of the *cis* configuration about the Leu-9–Leu-10 amide bond. In polar solvents, the conformational ensemble of cyclosporine A is more complex, with three to eight equilibrating conformations being proposed by NMR studies.^50^ One of these is the DEKSAN-type conformation, although with its population being reduced to 20–30%. Another conformation, often referred to as the 2Z6W-type (PDB code 2Z6W,^55^ Figure 8), has been suggested to be populated in polar solutions, but has not yet been experimentally proven.^50^ This conformation is adopted by cyclosporin A in protein complexes with cyclophilin A, B, and D. In contrast to the DEKSAN-type conformer, none of the amide protons of the 2Z6W-type form intramolecular hydrogen bonds, and its Leu-9–Leu-10 amide bond adopts a *trans* configuration. Consequently, the 2Z6W-type conformation is expected to be well-solvated via the formation of multiple intermolecular hydrogen bonds in an aqueous environment.^19^

**Figure 8:**
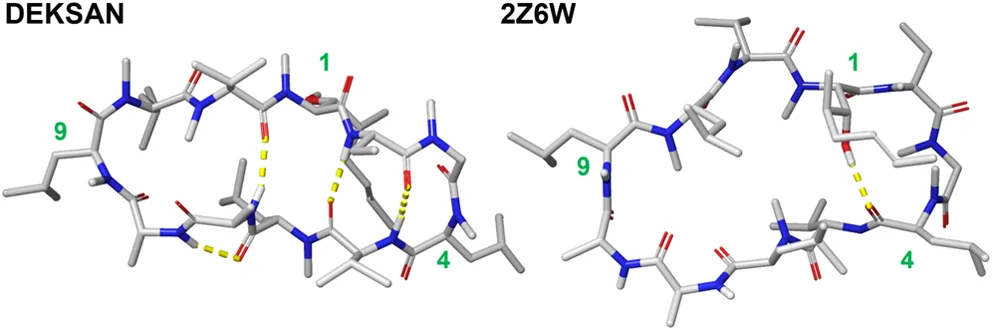
Two representative conformations of cyclosporine A. The closed, DEKSAN^53^-type conformation constitutes the major conformation in an apolar environment,^50^ while open conformations, such as 2Z6W,^55^ are predominant in a polar environment.^50^

Due to the ability to adjust its conformations, and thereby polarity, to the surrounding media, cyclosporine A is often used as a blueprint for a molecular chameleon.^18^ Griselimycin behaves in the opposite manner. Its conformational ensemble is more polar in an apolar environment than in a polar one. In an apolar environment, this conformational behavior appears to be driven by the exposure of griselimycin′s apolar amino-acid side chains and its pronounced degree of amide *N*-alkylation, instead of by intramolecular interactions that shield its polar moieties from the surrounding media. In aqueous solution, the apolar side chains undergo a hydrophobic collapse, simultaneously burying the polar moieties. Such behavior was recently reported for a few PROTACs,^29,57^ but was not previously considered in the field of molecular chameleons. We propose that compounds, such as griselimycin, for which hydrophobic intra- versus intermolecular interactions with the surrounding environment direct the conformational behavior, are named “*inverse chameleons*”. We conclude that inverse chameleonicity results in lowered cell permeability when compared to structurally similar chameleonic compounds. Just as for griselimycin and cyclosporine A, a PROTAC behaving as an inverse molecular chameleon had a substantially reduced permeability (20-fold in a cellular assay, close to 1000-fold in the PAMPA assay) as compared to its chameleonic structural analogue.^29^

## Summary and Conclusions

We found that the antibiotic macrocyclic peptide griselimycin has a low permeability in the PAMPA assay and across Caco-2 cell monolayers, and that its efflux rate is high (Figure 7). The solubility of griselimycin is low in biologically relevant aqueous media. Determination of the conformational ensembles of griselimycin, including its dipeptidic tail, using NMR spectroscopy in polar (D_2_O:DMSO-*d*_6_; 4:1) and apolar (CDCl_3_) solutions provided the structural rational for griselimycin′s low solubility and cell permeability.

Griselimycin displayed an atypical conformational behavior, previously reported only for a few PROTACs, when transitioning from an aqueous solution into an apolar, membrane-mimicking environment. Thus, we found the conformations of griselimycin to be less extended and to expose a smaller solvent-accessible 3D polar surface area in an aqueous environment than in an apolar one. This is suggested to arise from the fact that the apolar amino-acid side chains undergo a hydrophobic collapse in aqueous solution, which simultaneously buries the polar moieties of the peptide backbone. Conversely, solvation of the apolar side chains in an apolar environment necessitates the simultaneous exposure of the polar moieties. In contrast to molecular chameleons, griselimycin is thus unable to shield its polar moieties in an apolar environment. Instead, it adapts by optimization of its hydrophobic interactions with an apolar environment, i.e., it behaves as an “inverse molecular chameleon”. This inverse chameleonic behavior explains the lower cell permeability of griselimycin as compared to cyclosporine A, the prototypical molecular chameleon.

### Experimental Section

Griselimycin was purchased via BioNordika from Cayman Chemical (product nr. 39306, batch nr. 0687855–2) and had a purity of ≥98%. It was used without any further purification. NMR solvents were at a minimum reaction grade.

#### In Vitro Assays

The solubilities in phosphate-buffered saline (PBS), fasted-state simulated intestinal fluid (FaSSIF), fed-state simulated intestinal fluid (FeSSIF), and simulated gastric fluid (SGF), as well as the permeabilities in the PAMPA and Caco-2 assays of griselimycin and cyclosporin A, were determined at Cyprotex. The experimental polar surface area (EPSA) of griselimycin was determined at Cyprotex.

#### NMR Spectroscopy

NMR spectra were recorded for CDCl_3_ and D_2_O:DMSO-*d*_6_ (4:1) solutions at 25 °C on an 800 MHz Bruker Avance Neo NMR spectrometer equipped with a 5 mm Z-gradient TCI cryogenic probe. ^1^H, ^13^C, TOCSY, COSY, NOESY, HSQC, and HMBC spectra were used for assignment. EASY-ROESY spectra were recorded for a D_2_O:DMSO-*d*_6_ (4:1) solution on a Bruker Avance Neo 600 MHz spectrometer equipped with a 5 mm TCI cryogenic probe. Variable temperature NMR was recorded on a Bruker Avance Neo 500 MHz spectrometer equipped with a TXI cryogenic probe. Chemical shifts were referenced to TMS, indirectly via the solvent residual signal. ^1^H chemical shift assignments are listed in Tables S1–S2, Supporting Information. NOESY experiments were recorded using the noesyph pulse sequence^58,59^ with the mixing times 100, 200, 300, 400, 500, 600, and 700 ms, with a 2.5 s relaxation delay, and without solvent suppression. EASY-ROESY^38^ spectra were recorded using the roesyadesjsph^38,60–62^ pulse sequence with 50, 100, 150, 200, 250, 300, and 350 ms mixing times, with 2.5 s relaxation delay, and without solvent suppression. NMR spectra were processed using the software MestReNova (Version 15.1.0). NOE/ROE peak intensities were normalized according to $\sqrt{\frac{\mathrm{cross}\;\mathrm{peak}\;\mathrm{AB}\times \mathrm{cross}\;\mathrm{peak}\;\mathrm{BA}}{\mathrm{diagonal}\;\mathrm{peak}\;A\times \mathrm{diagonal}\;\mathrm{peak}\;B}}$,^63,64^ and interproton distances were estimated using the initial rate approximation.^63^ The build-up rates (σ_AB_) were calculated from at least five mixing times with linear regression *R* ^2^ ≥ 0.9. Interproton distances were calculated using the equation $r_{AB}=r_{ref}{\left(\frac{\sigma_{ref}}{\sigma_{AB}}\right)}^{1/6}$, where *r*_AB_ is the distance of interest, whereas *r*_ref_ is the known interatomic distance (1.78 Å) of the reference proton methylene pair and σ_ref_ is its build-up rate (Tables S3–S5, Supporting Information).

#### Computational Conformational Search

A theoretical ensemble of griselimycin was generated using unrestrained Monte Carlo Multiple Minima (MCMM) conformational search. All amide bonds but Pro-8 were restricted into *trans* configuration, whereas that of Pro-8 was defined as *cis* (ω = 0 ± 40°). The backbone conformations were identified first, using the MCMM output conformations. Next, the NAMFIS-identified macrocycle conformations were restricted (±0°), and the Val-1 - MePro-2 “tail” was allowed to rotate freely for each macrocycle geometry. The OPLS4, OPLS-2005, MMFF, and AMBER* force fields were used, each in combination with water and chloroform implicit solvent models. 100,000 Monte Carlo steps were performed for each conformational search, followed by a maximum of 5000 molecular mechanics minimization steps using the Polak–Ribiere conjugate gradient scheme (PCRG), as provided by the BatchMin algorithm of MacroModel (Schrödinger Release 2023–2: MacroModel, Schrödinger, LLC, New York, NY, 2023).^65,66^ Conformations within a 42 kJ/mol energy window from the global minimum were retained. The conformations from the 8 MCMM conformational searches were combined, and redundant conformations were eliminated (RCE) with a 0.9 Å RMSD cutoff, to obtain an input ensemble for NAMFIS. RCE was performed for the heavy atoms of the macrocycle in the first MCMM search, and subsequent to the Val-1 - MePro-2 “tail” only.

#### NAMFIS Analyses

The conformational ensembles of griselimycin were determined using the NAMFIS algorithm,^34^ by fitting population-weighted, back-calculated interproton distances and scalar couplings of theoretically feasible conformations to those experimentally observed. Methyl signals were averaged as d = (((d_1_^–6^)+ (d_2_^–6^)+ (d_3_^–6^))/3)^−1/6^ for spectra obtain for CDCl_3_ solution, and as d = (((d_1_^–3^)+ (d_2_^–3^)+ (d_3_^–3^))/3)^−1/3^ for those obtained for D_2_O:DMSO-*d*_6_ solutions, the reason for the different treatment being the different tumbling time due to different solvent viscosity. Overlapping peaks were treated as a single population-averaged entity, thus d = (((d_1_^–6^)+ (d_2_^–6^)+···+[(d_*x*_^–6^)]))^−1/6^.^67^ Diastereotopic methylene protons were identified based on RMSD in the NAMFIS outputs. Scalar couplings for the theoretically predicted conformers were back-calculated from dihedral angles ω using a Karplus equation developed for peptides (eq S1, Supporting Information).^68^ Depending on the type of protons, different parameters for the Karplus Equation were necessary (Table S8).^69,70^ NAMFIS results were validated by the Jackknife test and by adding ±5% random noise to the experimental distances, resulting in < ±10% alteration of the population of conformational families.

#### Amide Temperature Coefficients

[Δδ/Δ*T*] (ppb/K) were measured for CDCl_3_ and H_2_O:DMSO-*d*_6_ (4:1) solutions of griselimycin by detecting ^1^H NMR spectra at temperatures 20 to 45 °C, in 5 °C steps.

#### Molecular Descriptor Calculations

The radius of gyration (*R*_gyr_), the solvent-accessible surface area (SA SA), and the solvent-accessible 3D polar surface area (SA 3D PSA) were calculated using VEGA ZZ (Release 3.2.3).^71^ The solvent-accessible nonpolar surface area (SA 3D NPSA) was calculated by subtracting the SA 3D PSA from the SA SA.

This research did not involve human or animal participants.

## Supplementary Material





## Data Availability

The data that support the findings of this study (original NMR FIDs, MNova files, NMReDATA, NAMFIS files, mol2 structure files) are freely available at the open access repository Zenodo via 10.5281/zenodo.19458261.

## Notes

Molecular formula string (CSV)
Experimental details of NMR data acquisition and data processing, NAMFIS analyses, and calculation of molecular descriptors (PDF)

## Abbreviations

bRo5: beyond the rule of five
CsA: cyclosporine A
EASY-ROESY: efficient adiabatic symmetrized rotating overhauser effect spectroscopy
EPSA: experimental polar surface area
ER: efflux ratio
IMHB: intra molecular hydrogen bond
MCMM: Monte Carlo multiple minima
NAMFIS: NMR analysis of molecular flexibility in solution
PROTAC: proteolysis targeting chimera
RCE: redundant conformer elimination
R_gyr_: radius of gyration
SA 3D NPSA: solvent-accessible 3D nonpolar surface area
SA 3D PSA: solvent-accessible 3D polar surface area
SA SA: solvent-accessible 3D surface area
SGF: simulated gastric fluid
VT-NMR: variable temperature NMR
