# Supplementary material for: Solution Ensembles of Griselimycin Expand the Definition of Molecular Chameleonicity
Source: J Med Chem. 2026 Jul 21;69(15):19219–29. doi: 10.1021/acs.jmedchem.6c01558 (PMC13492371; doi:10.1021/acs.jmedchem.6c01558)
Supplement: Supplementary file 1 [file jm6c01558_si_002.pdf]

Supporting information

The Solution Ensembles of Griselimycin Expand the Definition of Molecular Chameleonicity

Luca J. Hagemeyer,[†] William T. P. Darling,[†, Δ,] Nele-Johanna Hempel-Højgaard,[‡] Jan Kihlberg,[†,*] Mate Erdelyi[†, Δ,*]

[†] Department of Chemistry for Life Sciences, Uppsala University, Uppsala SE 751 23, Sweden

‡ Novo Nordisk A/S, Therapeutics Discovery, Research & Development, Novo Nordisk Park 1, 2760 Måløv, Denmark

[Δ] Center of Excellence for the Chemical Mechanisms of Life, Uppsala University, Uppsala SE 751 23, Sweden

*jan.kihlberg@kemi.uu.se (J.K.), mate.erdelyi@kemi.uu.se (M.E.)

Table of Contents

1. ^{1}H NMR assignments S3

2. Assignment for $CDCl_3$ solution S4

3. Assignment for D_2O:DMSO-d_6 (4:1) solution S5

4. Experimental data S7

4.1. NOE/ROE build-up rates and interproton distances S7

4.1.1. NOESY for $CDCl_3$ solution S7

4.1.2. NOESY build-up rates for D_2O:DMSO-d_6 (4:1) solution S8

4.1.3. ROESY-build-up rates for D_2O:DMSO-d_6 (4:1) solution S9

4.1.4 Scalar-couplings S10

4.1.5 Amide temperature coefficients S11

5. Monte-Carlo Molecular Mechanics (MCMM) conformational search parameters S11

6. NAMFIS analyses S13

6.1 Conformers identified for the $CDCl_3$ solution using NAMFIS S13

6.2 Conformers identified for the D_2O/DMSO-d_6 solution using NAMFIS, based on NOESY build-up data S18

6.3. Conformers identified for the D_2O/DMSO-d_6 solution using NAMFIS, based on ROESY data S21

7. Molecular descriptors S25

7.1 Calculated descriptors for conformational ensemble in $CDCl_3$ solution[3] S25

7.2 Calculated descriptors for ensemble in D_2O:DMSO-d_6 (4:1) solution, based on ROESY data[3] S25

7.3 Experimental polar surface area (EPSA) S26

8. References S26

Original NMR FIDs, structure files (pdb) and NAMFIS files are freely available online at Zenodo via https://doi.org/10.5281/zenodo.19458261

1. ^{1}H NMR assignments

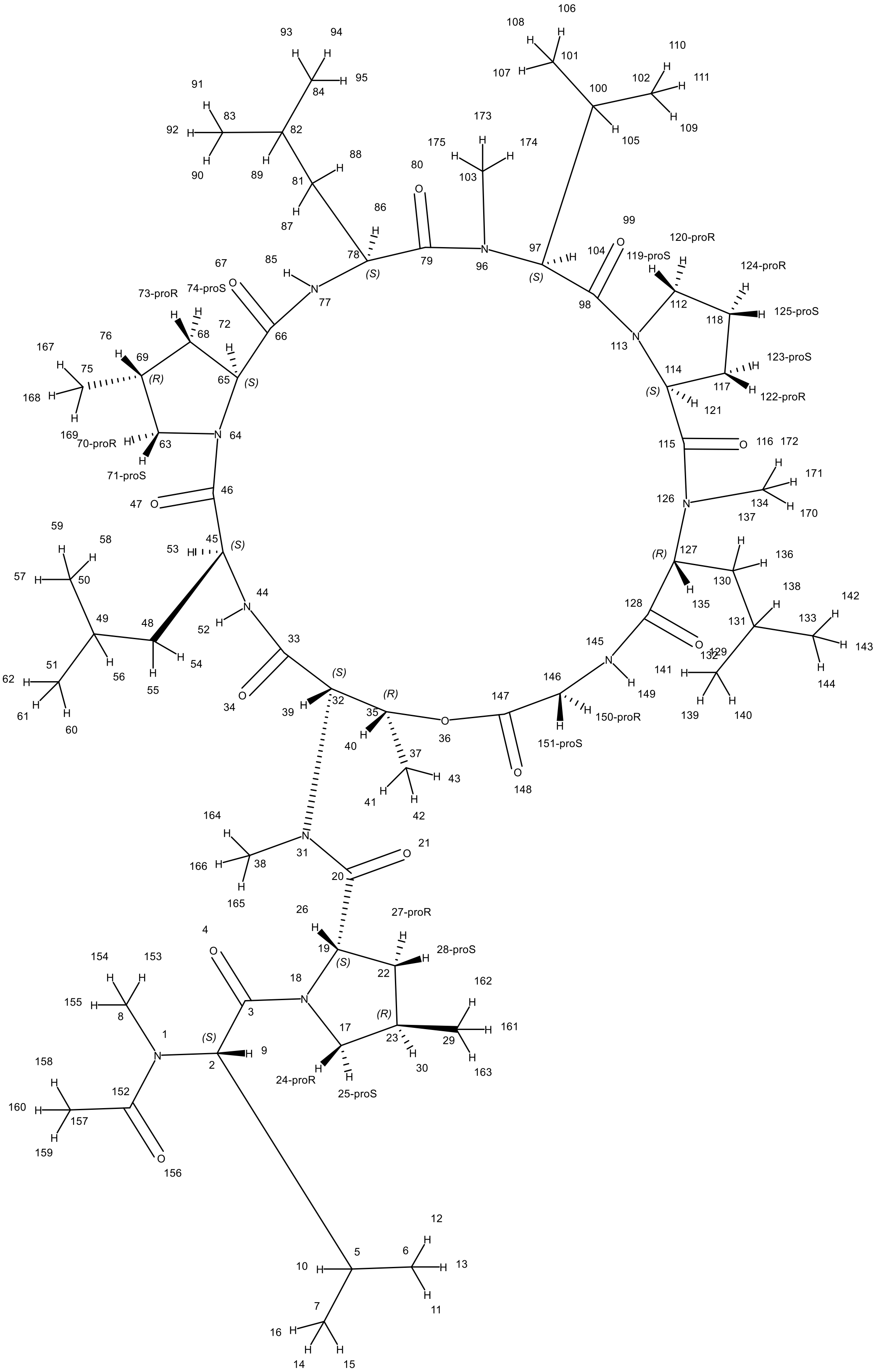

Figure S1. *Griselimycin showing atom numbering and stereo descriptors.*

2. Assignment for $CDCl_3$ solution

Table S1. ^{1}H NMR assignment of griselimycin in $CDCl_3$ solution

Chemical shift [ppm]	Atom number	Type
8.81	149	^{10}Gly-NH
7.84	85	^{6}Leu-NH
6.48	52	^{4}Leu-NH
5.93	40	^{3}Thr-βH
5.61	39	^{3}Thr-αH
5.11	135	^{9}Leu-αH
5.07	9	^{1}Val-αH
4.99	86	^{6}Leu-αH
4.94	26	^{2}MePro-αH
4.77	121,<br>104	^{8}Pro-αH<br>^{7}Val-αH
4.60	72	^{5}MePro-αH
4.38	53	^{4}Leu-αH
4.23	150	^{10}Gly-αH
4.06	151	^{10}Gly-αH
3.96	25	^{2}MePro-δH
3.73	120	^{8}Pro-δH
3.60	119	^{8}Pro-δH
3.50	70	^{4}MePro-δH
3.37	24	^{2}MePro-δH
3.30	173-175	^{7}Val-NMe
3.12	71,<br>164-166	^{5}MePro-αH<br>^{3}Thr-NMe
3.03	153-155	^{1}Val-NMe
2.88	170-172	^{9}Leu-NMe
2.62	30	^{2}MePro-γH
2.57	73	^{5}MePro-βH
2.43	76	^{5}MePro-γH
2.34	123	^{8}Pro-βH
2.25	105,<br>10	^{7}Val-βH<br>^{1}Val-βH
2.11	27,<br>158-160	^{2}MePro-βH<br>^{1}Val-Ac
1.98	122	^{8}Pro-βH
1.90	124,<br>137	^{8}Pro-γH<br>^{9}Leu-βH
1.84	125	^{8}Pro-γH
1.77	28,<br>56	^{2}MePro-βH<br>^{4}Leu-γH
1.51	87, 88	^{6}Leu-βH
1.48	55	^{4}Leu-βH
1.46	138	^{9}Leu-γH
1.43	89	^{6}Leu-γH

1.41	74	[5]MePro-βH
1.35	41-43	[3]Thr-CH_3
1.27	136,<br>54	[9]Leu-βH<br>[4]Leu-βH
1.08	167-169	[5]MePro-CH_3
1.01	161-163	[2]MePro-CH_3
0.99	11-13	[1]Val-CH_3
0.95	57-59, 60-62	[4]Leu-CH_3
0.92	139-141, 142-144	[9]Leu-CH_3
0.91	90-92, 93-95	[6]Leu-CH_3
0.89	109-111	[7]Val-CH_3
0.85	14-16	[1]Val-CH_3
0.76	106-108	[7]Val-CH_3

3. Assignment for D_2O:DMSO-*d*$_6$ (4:1) solution

Table S2. ^{1}H NMR assignment of griselimycin in D_2O:DMSO-*d*$_6$ (4:1) solution

Chemical shift [ppm]	Atom number	Type
8.84	85	[6]Leu-NH[a]
8.31	149	[10]Gly-NH[a]
7.83	52	[4]Leu-NH[a]
5.73	40	[3]Thr-βH
5.28	39	[3]Thr-αH
5.06	121	[8]Pro-αH
5.02	135	[9]Leu-αH
4.81	26	[2]MePro-αH
4.76	9	[1]Val-αH
4.71	104	[7]Val-αH
4.55	86	[6]Leu-αH
4.51	72	[5]MePro-αH
4.16	53	[4]Leu-αH
4.12	150	[10]Gly-αH
3.91	151	[10]Gly-αH
3.65	25	[2]MePro-δH
3.47	71	[5]MePro-δH
3.39	119,<br>120	[8]Pro-δH<br>[8]Pro-δH
3.16	164-166	[3]Thr-NMe
3.08	173-175	[7]Val-NMe
3.05	24	[2]MePro-δH
3.01	70	[5]MePro-δH
2.79	153-155	[1]Val-NMe
2.76	170-172	[9]Leu-NMe

[a] Recorded in H_2O/DMSO-*d*$_6$ (4:1) at 500 MHz

2.28	30	[2]MePro-γH
2.26	123	[8]Pro-βH
2.18	73*	[5]MePro-βH
2.14	76	[5]MePro-γH
2.05	105,<br>10	[7]Val-βH<br>[1]Val-βH
2.00	158-160	[1]Val-Ac
1.91	122	[8]Pro-βH
1.83	27,<br>28,<br>124*	[2]MePro-βH<br>[2]MePro-βH<br>[8]Pro-γH
1.61	56	[4]Leu-γH
1.54	125*,<br>136*	[8]Pro-γH,<br>[9]Leu-βH
1.38	74*,<br>54*	[5]MePro-βH<br>[4]Leu-βH
1.32	137*	[8]Pro-γH
1.30	87,<br>88,<br>89	[6]Leu-βH<br>[6]Leu-βH<br>[6]Leu-γH
1.26	55*	[4]Leu-βH
1.16	138,<br>41-43	[9]Leu-γH<br>[3]Thr-CH_3
0.90	167-169	[5]MePro-CH_3
0.83	161-163	[2]MePro-CH_3
0.78	57-59,<br>60-62	[4]Leu-CH_3<br>[4]Leu-CH_3
0.76	93-95,<br>142-144	[6]Leu-CH_3<br>[9]Leu-CH_3
0.72	14-16,<br>109-111	[1]Val-CH_3<br>[7]Val-CH_3
0.70	139-141	[9]Leu-CH_3
0.69	90-92	[6]Leu-CH_3
0.65	11-13	[1]Val-CH_3
0.59	106-108	[7]Val-CH_3

*Methylene protons not distinguishable

4. Experimental data

4.1. NOE/ROE build-up rates and interproton distances

4.1.1. NOESY for $CDCl_3$ solution

Table S3. NOE build-up table for $CDCl_3$ solution (800 MHz)

No	Proton A	Proton B	Build-up rate σ * 10^6	R^2	Distance r_{AB} [Å]
1	149	85	4.484	0.99	3.26
2	149	135	18.333	1.00	2.58
3	85	52	1.277	0.94	4.02
4	85	72	14.625	1.00	2.68
5	52	53	5.479	0.95	3.15
6	52	39	13.178	1.00	2.73
7	52	40	60.646	1.00	2.11
8	40	39	32.778	1.00	2.34
9	39	53	1.471	0.97	3.93
10	85	86	13.520	0.99	2.71
11	86	72	19.969	1.00	4.28
12	149	150	69.890	0.99	2.17
13	149	151	22.213	0.95	2.50
14	85	151	0.393	0.92	4.44
15	85	150	6.651	1.00	3.05
16	52	150	0.298	0.95	5.13
17	40	151	0.656	0.96	4.49
18	53	76	7.406	0.99	4.29
19	53	70	29.399	1.00	2.38
20	151	41-43	1.182	0.94	4.07
21	85	173-175	0.972	0.98	4.21
22	86	173-175	4.205	0.99	2.54
23	149	170-172	1.671	0.95	3.84
24	135	170-172	6.177	0.99	4.60
25	170-172	123	2.595	0.98	2.91
26	170-172	122	7.739	0.98	2.54
27	72	73	1.203	0.96	2.84
28	149	121,104	2.976	0.97	3.49
29	121,104	123	10.184	0.98	2.70
30	121,104	122	0.873	0.94	3.69
31	121,104	119	18.652	1.00	3.51
32	121,104	120	2.863	0.95	3.96
33	9	25	3.226	0.96	3.09
34	9	24	0.881	0.94	3.45

35	26	30	3.888	1.00	4.91
36	39	71,164-166	0.572	0.96	4.28
37	26	71,164-166	0.386	0.96	2.19
38	72	71,164-166	11.697	1.00	4.06
39	53	71,164-166	7.469	0.97	3.00
40	151	71,164-166	4.038	0.92	3.86
41	150	71,164-166	3.221	0.99	3.45
42	39	30	1.118	0.94	4.11
43	39	28,56	0.950	0.96	4.24
44	153-155	27,158-160	60.788	0.93	2.91
Ref	70	71	169.781	1.00	1.78

4.1.2. NOESY build-up rates for D_2O:DMSO-d_6 (4:1) solution

Table S4. NOE build-up table for D_2O:DMSO-d_6 (4:1) solution (800 MHz)

No	Proton A	Proton B	Build-up rate σ * 10^6	R^2	Distance r_{AB} [Å]
1	40	39	438.916	0.99	2.23
2	39	53	8.729	0.92	4.28
3	121	104	646.470	1.00	2.09
4	121	135	5.090	0.93	4.68
5	135	170-172	33.739	1.00	3.41
6	121	170-172	250.611	1.00	2.44
7	104	170-172	74.733	0.99	2.99
8	135	173-175	3.783	0.91	4.91
9	121	173-175	12.025	0.95	4.05
10	104	173-175	29.160	0.98	3.50
11	72	76	29.582	0.94	3.49
12	86	173-175	311.961	0.98	2.36
13	121	105,10	18.836	0.98	3.76
14	170-172	173-175	3.254	0.96	5.04
15	72	167-169	19.274	0.94	3.75
16	40	164-166	168.562	1.00	2.61
17	39	164-166	113.086	1.00	2.79
18	39	164-166	39.358	1.00	3.33
19	40	164-166	29.820	0.97	3.48
20	164-166	138,41-43	51.909	1.00	3.18
21	135	151	5.221	0.91	4.66
22	40	150	16.929	0.98	3.83
23	150	164-166	26.568	0.97	3.55
24	150	170-172	15.892	0.99	3.87
25	53	70	420.233	1.00	2.24

26	135	119,120	3.837	0.99	4.9
28	72	150	57.232	0.99	3.12
27	135	122	11.275	0.98	4.10
29	104	119,120	18.142	0.92	3.78
30	119,120	173-175	9.404	0.92	4.22
31	170-172	122	199.517	1.00	2.54
32	26	164-166	309.870	1.00	2.36
33	26	153-155	5.350	0.97	4.64
34	9	153-155	17.232	0.96	3.82
35	9	24	202.665	0.99	2.53
36	9	25	252.403	0.99	2.44
37	26	24	48.231	0.95	3.22
38	26	25	12.833	0.96	4.01
39	26	161-163	9.996	0.96	4.18
40	26	41-43,138	19.669	0.99	3.73
41	25	158-160	10.891	0.93	4.12
42	25	164-166	16.570	0.97	3.84
43	25	153-155	8.758	0.97	4.27
44	25	161-163	33.605	0.99	3.41
45	24	153-155	13.423	0.99	3.98
46	24	161-163	35.975	1.00	3.38
47	24	158-160	9.613	0.96	4.21
48	164-166	161-163	7.987	0.99	4.34
49	153-155	158-160	109.017	1.00	2.81
50	158-160	161-163	3.129	0.98	5.07
51	39	26	19.821	0.99	3.73
52	39	30	5.118	0.97	4.67
53	164-166	30	11.821	0.99	4.06
Ref	40	41-43,138	168.562	1.00	2.61

4.1.3. ROESY-build up rates for D_2O:DMSO-d_6 (4:1) solution

Table S5. ROE build-up table for D_2O:DMSO-d_6 (4:1) solution (600 MHz)

No	Proton A	Proton B	Build-up rate σ * 10^6	R^2	Distance r_{AB} [Å]
1	40	39	241.793	1.00	2.27
2	121	104	407.343	1.00	2.08
3	135	170-172	8.486	0.99	3.97
4	121	170-172	70.156	1.00	2.79
5	104	173-175	14.495	0.98	3.63
6	40	138,41-43	104.654	1.00	2.61

7	39	138,41-43	48.218	1.00	2.97
8	39	164-166	13.039	0.99	3.69
9	40	164-166	4.670	0.97	4.38
10	164-166	138,41-43	26.026	1.00	3.29
11	53,151	70,25	57.303	1.00	2.89
12	104	119,120	4.585	0.91	4.40
13	53,151	71	56.210	0.96	2.89
14	150	138,41-43	5.031	0.96	4.33
15	150	164-166	9.536	0.94	3.89
16	150	170-172	9.858	0.97	3.87
17	71	167-169	11.418	0.98	3.78
18	173-175	105,10	53.876	1.00	2.92
19	26	164-166	172.696	1.00	2.40
20	9	70,25	76.327	1.00	2.75
21	9	24	179.039	1.00	2.39
22	26	70,25	27.782	1.00	3.26
23	26	138,41-43	3.229	0.98	4.66
24	39	26	2.156	0.95	4.99
25	24	30,122,123	100.694	1.00	2.63
26	70,25	161-163	11.939	0.98	3.75
27	70,25	167-169	45.331	0.98	3.00
28	30,122,123	161-163	62.688	0.96	2.84
ref (No. 6)	40	138,41-43	104.654	1.00	2.61

4.1.4 Scalar-couplings

Table S6. $^3J_{HH}$-couplings griselimycin observed for $CDCl_3$ solution

No.	Proton A	Proton B	Measured J-coupling [Hz]
1	52	53	8.1
2	85	86	9.3

Table S7. $^3J_{HH}$-couplings griselimycin in H_2O:DMSO-d_6 (4:1) solution

No.	Proton A	Proton B	Measured J-coupling [Hz]
1	52	53	5.3
2	85	86	7.5
3	149	150	5.5
4	149	151	5.5
5	39	40	3.8

Scalar couplings for the computationally generated conformers were back-calculated from the dihedral angle (ω) using Karplus equations parametrized for specific bond types (eq. S1)

$$^{3}J_{HH} = A * \cos^{2}(\omega) + B * \cos(\omega) + C \qquad \text{(Equation S1)}$$

Table S8. Karplus-parameter for back-calculation of scalar couplings

	A	B	C
$^{3}J_{NH\text{-}\alpha H}$	9.40[1]	-1.10[1]	0.40[1]
$^{3}J_{\alpha H\text{-}\beta H}$(Thr)	7.23[2]	-1.37[2]	2.22[2]

4.1.5 Amide temperature coefficients

Table S9. Amide proton chemical shift temperature coefficients (ppb/K) observed for $CDCl_3$ and H_2O:DMSO-d_6 (4:1) solution for temperature ranges 15 and 40 °C, respectively.

	$CDCl_3$ [ppb/K]	H_2O:DMSO-d_6 (4:1) [ppb/K]
^{4}Leu-NH	-1.09	-9.49
^{6}Leu-NH	-1.09	-5.43
^{10}Gly-NH	-2.63	-3.54

In a polar protic solvent, a large, $\Delta\delta/\Delta T > 5$ ppb/K, reflects a solvent exposed amide proton, $\Delta\delta/\Delta T < 3$ ppb/K a strong intramolecular hydrogen bond that is not easily interrupted, whereas $\Delta\delta/\Delta T \sim 3$ -5 ppb/K an amide proton involved into an equilibrium between weak intra- and intermolecular hydrogen bonds. In an aprotic solvent, a $\Delta\delta/\Delta T < 3$ ppb/K indicates either a strong intramolecular hydrogen bond or a solvent exposed amide, whereas $\Delta\delta/\Delta T > 3$ ppb/K a proton in equilibrium between intra- and intermolecular hydrogen bonds.

5. Monte-Carlo Multiple Minima (MCMM) conformational search parameters

Table S10. MCMM griselimycin macrocyclic core

Residue	Implicit solvent model	Force field	Number of conformers[b]
Griselimycin $CDCl_3$ core	$CHCl_3$<br>H_2O	OPLS4<br>OPLS-2005<br>MMFF<br>AMBER*	59 (0.9 Å)[c]

[b]Distinct conformers with a 0.9 Å heavy atom RMSD cut-off

[c]Redundant conformer elimination based on the macrocyclic core atoms, β-carbons and *N*-alkyls (excluding ^{3}Thr-NMe)

Griselimycin<br>D_2O/DMSO<br>core	$CHCl_3$<br>H_2O	OPLS4<br>OPLS-2005<br>MMFF<br>AMBER*	142 (0.9 Å)[d]

Table S11. MCMM griselimycin dipeptidic sidechain for $CDCl_3$ solution

Residue	Implicit solvent (each conf.)	Force field (each conf.)	Core conformer (59)[e]	Number of conformers[f]	Total number of conformers
Griselimycin<br>$CDCl_3$<br>Full	$CHCl_3$<br>H_2O	OPLS4<br>OPLS-2005<br>MMFF<br>AMBER*	No. 2<br>No. 7<br>No. 13<br>No. 45<br>No. 46<br>No. 56<br>No. 57	38<br>17<br>26<br>48<br>56<br>50<br>13	248

Table S12. MCMM griselimycin dipeptidic sidechain for D_2O/DMSO-d_6 solution (NOESY)

Residue	Implicit solvent (each conf.)	Force field (each conf.)	Core conformer (142)[e]	Number of conformers[f]	Total number of conformers
Griselimycin<br>D_2O/DMSO<br>NOESY	$CHCl_3$<br>H_2O	OPLS4<br>OPLS-2005<br>MMFF<br>AMBER*	No. 32<br>No. 34<br>No. 35<br>No. 41<br>No. 42<br>No. 46<br>No. 62<br>No. 87<br>No. 117<br>No. 128	17<br>19<br>16<br>24<br>25<br>23<br>18<br>15<br>12<br>20	189

Table S13. MCMM griselimycin dipeptidic tail in D_2O/DMSO-d_6 (ROESY)

Residue	Implicit solvent (each conf.)	Force field (each conf.)	Core conformer (142)[e]	Number of conformers[f]	Total number of conformers

[d]Redundant conformer elimination based on the macrocyclic core atoms, β- carbons and *N*-alkyls (including [3]Thr-NMe)

[e] Numbers corresponding to the conformers originating from the theoretical ensemble used for the macrocycle core sampling. The size of the theoretical ensemble is indicated by the number in brackets

[f] Population of each macrocycle core conformer in the theoretical input ensemble used in NAMFIS

Griselimycin<br>D_2O/DMSO<br>ROESY	$CHCl_3$<br>H_2O	OPLS4<br>OPLS-2005<br>MMFF<br>AMBER*	No. 3<br>No. 21<br>No. 31<br>No. 35<br>No. 46<br>No. 110<br>No. 117	28<br>26<br>26<br>28<br>30<br>28<br>16	182

Macrocycle conformations were first identified and validated with NAMFIS. These conformations were subsequently subjected to further Monte Carlo conformational searches restricting any rotation of the bonds of the macrocyclic core, whereas the bond angles of the dipeptidic side chain were allowed to freely rotate. For each conformer, MCMM searches using two implicit solvent models and four different force fields were used. Redundant conformers from each simulation were eliminated, then the conformers were combined, and were used as the theoretical input ensemble for NAMFIS calculations.

6. NAMFIS analyses

6.1 Conformers identified for the $CDCl_3$ solution using NAMFIS

The conformers identified by NAMFIS for $CDCl_3$ solution, based on experimental NOE-derived inter-proton distances and scalar couplings.

Table S14. The populations of the macrocyclic core conformers identified by NAMFIS for the $CDCl_3$ solution of griselimycin

Conf. No.	Population (%)	Conformational family	Population (%)
$I^{g}_{CDCl3\_MC}$	9	A	77
$III^{g}_{CDCl3\_MC}$	15		
$IV^{g}_{CDCl3\_MC}$	7		
$V^{g}_{CDCl3\_MC}$	45		
$II^{g}_{CDCl3\_MC}$	6	B	20
$VI^{g}_{CDCl3\_MC}$	14		
$VII^{g}_{CDCl3\_MC}$	3	C	3

Table S15. The populations of the conformers identified by NAMFIS for the $CDCl_3$ solution of griselimycin

Conf. No.[h]	Population (%)	Conformational family	Population (%)
$\mathbf{7^{h}_{CDCl3\_full}}$	41	A1	41

[g] Roman numbering indicates macrocycle core conformers
[h] Arabic numbers indicate "griselimycin conformers, including also sidechain atoms

$\mathbf{1^{h}_{CDCl3\_full}}$	17	A2	25
$\mathbf{2^{h}_{CDCl3\_full}}$	8		
$\mathbf{5^{h}_{CDCl3\_full}}$	13	A3	13
$\mathbf{6^{h}_{CDCl3\_full}}$	8	A4	8
$\mathbf{3^{h}_{CDCl3\_full}}$	4	B1	12
$\mathbf{4^{h}_{CDCl3\_full}}$	8		

Table S16. NOE-based experimental and back-calculated distances (NAMFIS) for $CDCl_3$ solution

No	Proton A	Proton B	Experimental Distance	Back-calculated Distance
1	149	85	3.26	3.39
2	149	135	2.58	2.54
3	85	52	4.02	4
4	85	72	2.68	2.62
5	52	53	3.15	2.9
6	52	39	2.73	2.61
7	52	40	2.11	2.08
8	40	39	2.34	2.42
9	39	53	3.93	4.48
10	85	86	2.71	2.93
11	86	72	4.28	4.58
12	149	150	2.17	2.41
13	149	151	2.5	2.66
14	85	151	4.44	4.45
15	85	150	3.05	3.46
16	52	150	5.13	4.6
17	40	151	4.49	4.42
18	53	76	4.29	4.74
19	53	70	2.38	2.29
20	151	41-43	4.07	4.55
21	85	173-175	4.21	5.04
22	86	173-175	2.54	2.41
23	149	170-172	3.84	3.82
24	135	170-172	4.6	3.87
25	170-172	123	2.91	3.41
26	170-172	122	2.54	2.72
27	72	73	2.84	2.82
28	149	121,104	3.49	3.47
29	121,104	123	2.7	2.35
30	121,104	122	3.69	2.75

31	121,104	119	3.51	3.81
32	121,104	120	3.96	3.46
33	9	25	3.03	2.93
34	9	24	3.45	3.31
35	26	30	4.91	3.94
36	39	71,164-166	4.28	3.85
37	26	71,164-166	2.23	2.37
38	72	71,164-166	4.15	3.92
39	53	71,164-166	3.07	2.65
40	151	7,164-166	3.86	4.08
41	150	71,164-166	3.63	4.18
42	39	30	4.11	4.7
43	39	28,56	4.22	4.33
44	153-155	27,158-160	2.91	2.83

Table S17. Experimental and NAMFIS-derived back-calculated scalar couplings for $CDCl_3$ solution

No	Proton A	Proton B	Experimental $^3J_{HH}$	Back-calculated $^3J_{HH}$
1	52	53	7.8	8.3
2	85	86	9.3	8.8

Table S18. The RMSD values comparing the positions of heavy atoms of the peptide backbone of griselimycin conformers, identified for $CDCl_3$ solution by NAMFIS. A lower RMSD value indicates higher similarity.

	1_A2	2_A2	3_B1	4_B1	5_A3	6_A4	7_A1
1_A2	0						
2_A2	1.237	0					
3_B1	2.719	2.275	0				
4_B1	2.404	2.287	1.003	0			
5_A3	2.295	2.321	3.483	3.416	0		
6_A4	1.385	1.712	3.32	3.051	2.153	0	
7_A1	1.97	1.589	2.618	2.765	2.151	2.16	0

Table S19. Populated conformers of griselimycin in $CDCl_3$ solution (based on NOESY). Graphical representation including intramolecular hydrogen bonds. Conformers (pdb) are provided on Zenodo via https://doi.org/10.5281/zenodo.19458261.

No	Conf	Conformational family	Population [%]
7 **CDCl3_full**	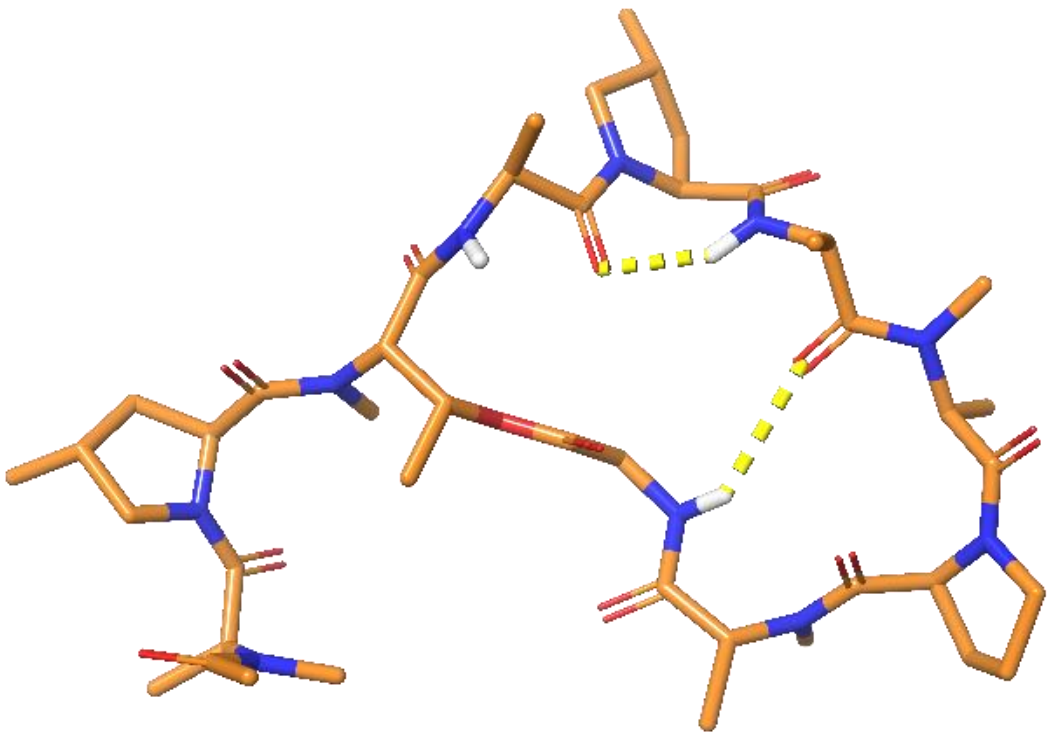	A1	41
1 **CDCl3_full**	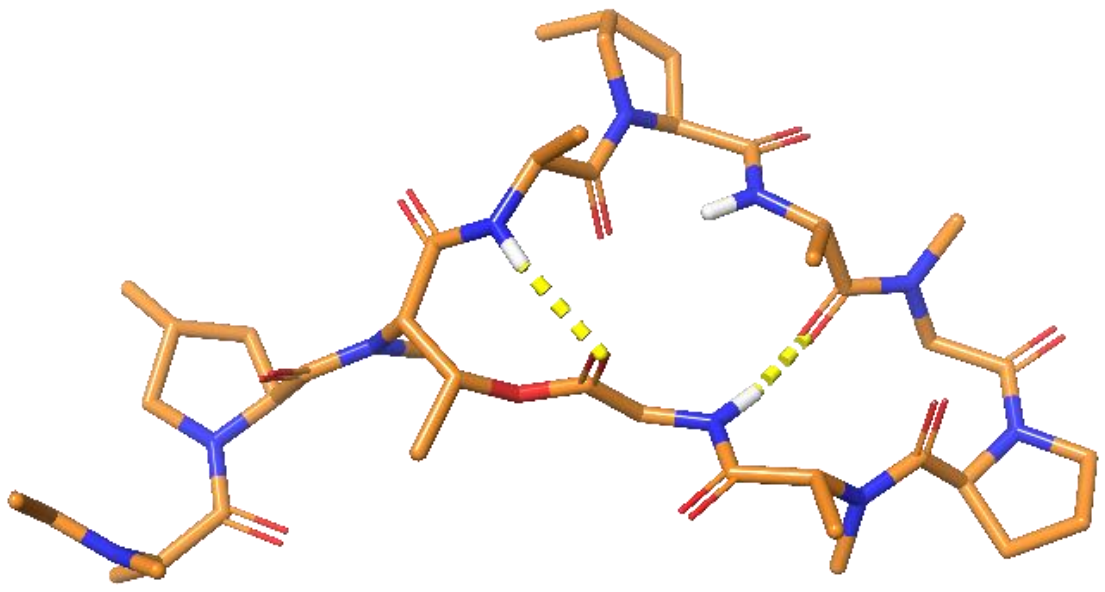	A2	17
2 **CDCl3_full**	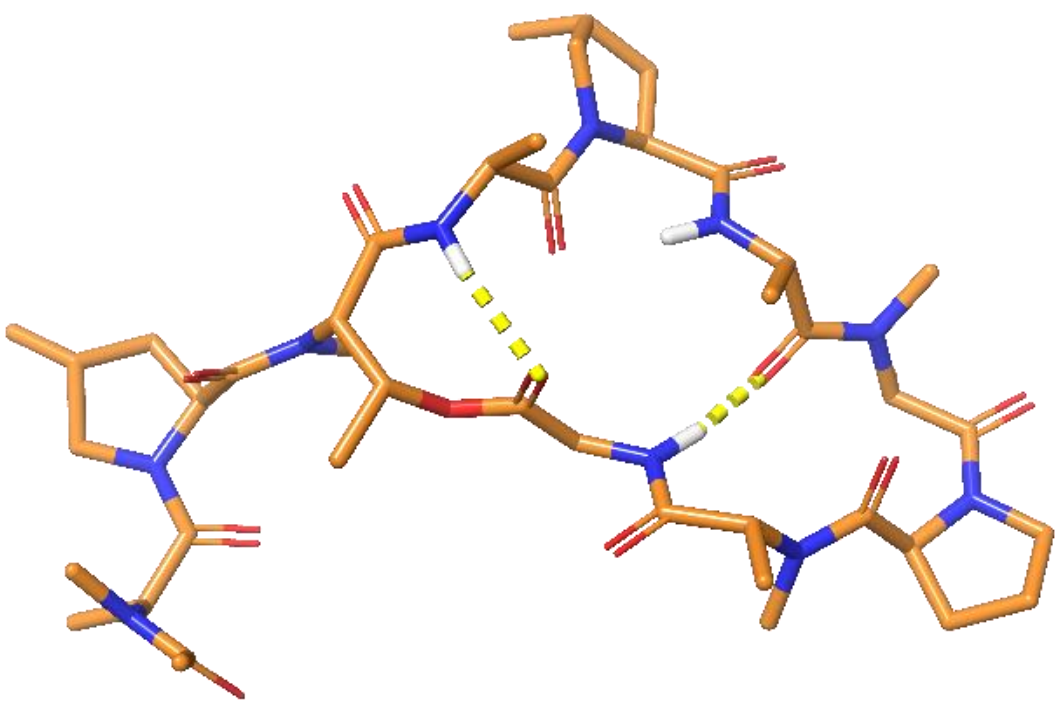	A2	8
5 **CDCl3_full**	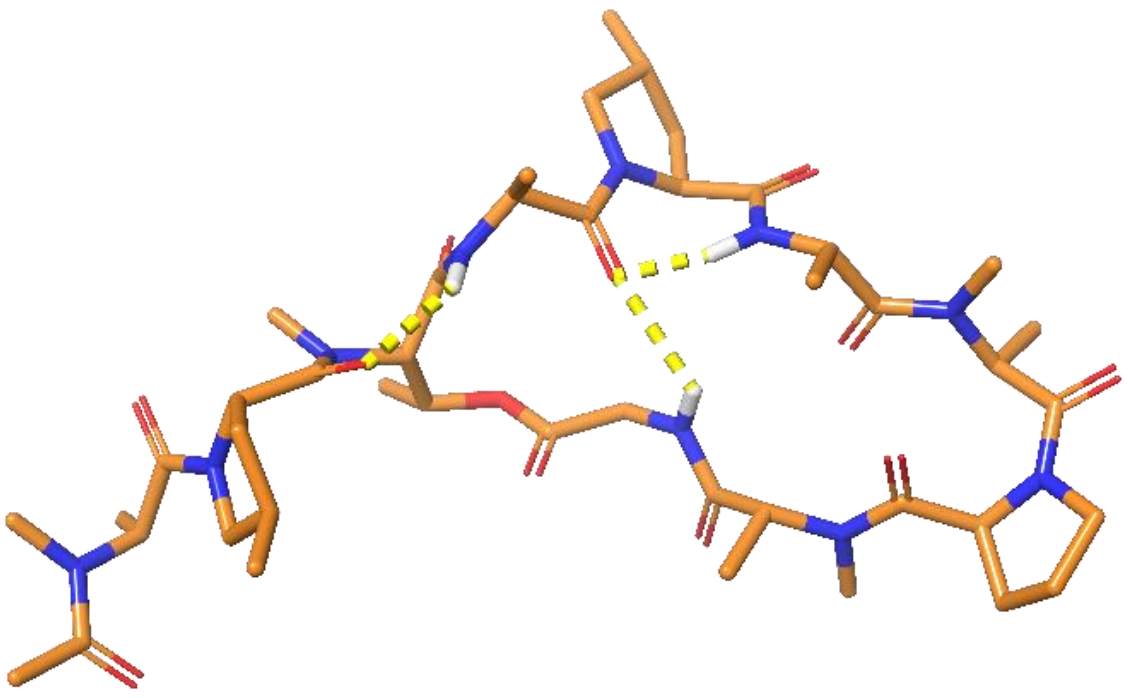	A3	13

6 **CDCl3_full**	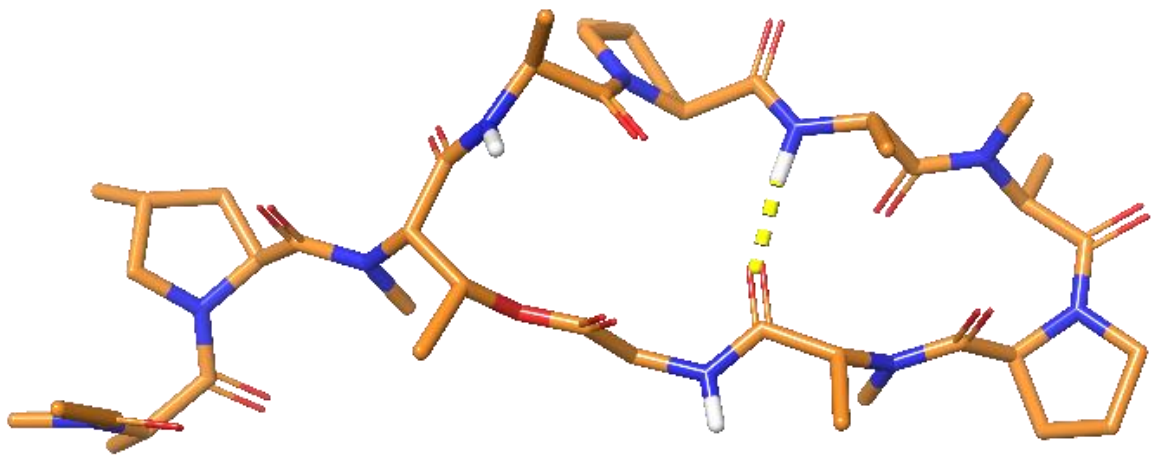	A4	8
3 **CDCl3_full**	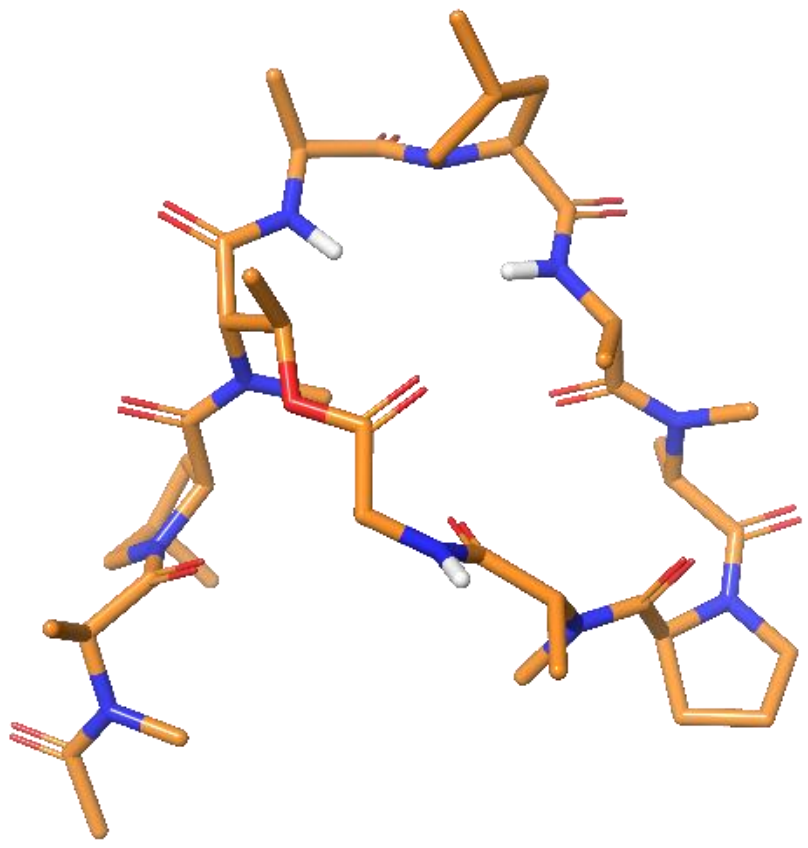	B1	4
4 **CDCl3_full**	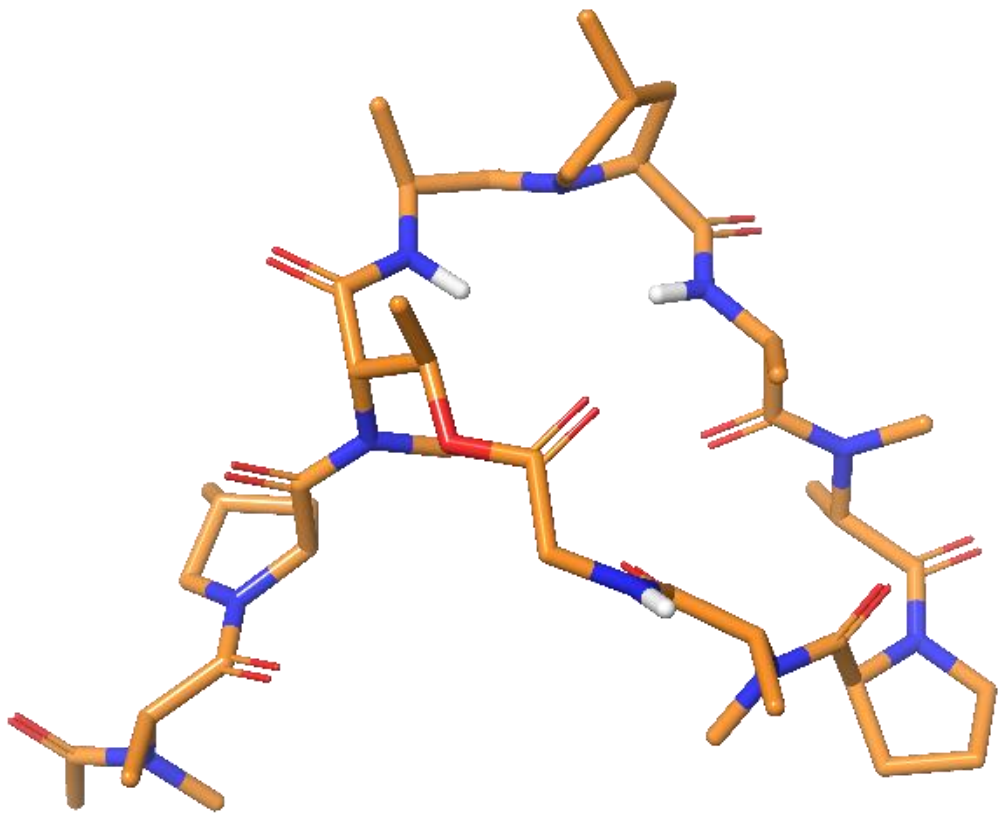	B1	8

6.2 Conformers identified for the D_2O/DMSO-d_6 solution using NAMFIS, based on NOESY build-up data

This data could not be validated, and therefore the data derived from ROESY build-up is reported in the main text. We provide this data, however, for completeness, for users interested in comparing NOESY- and ROESY-build-up based datasets

Table S20. Conformers identified using NAMFIS for the macrocyclic core of griselimycin in D_2O:DMSO-d_6 (4:1) solution, based on NOESY

Conf. No.	Population (%)	Conformational family	Population (%)
I[i] $_{\text{D2ODMSO\_MC\_NOE}}$	7	A	66[j]
IV[i] $_{\text{D2ODMSO\_MC\_NOE}}$	14		
V[i] $_{\text{D2ODMSO\_MC\_NOE}}$	17		
VII[i] $_{\text{D2ODMSO\_MC\_NOE}}$	3		
VIII[i] $_{\text{D2ODMSO\_MC\_NOE}}$	8		
II[i] $_{\text{D2ODMSO\_MC\_NOE}}$	7	B	27[h]
III[i] $_{\text{D2ODMSO\_MC\_NOE}}$	21		
VI[i] $_{\text{D2ODMSO\_MC\_NOE}}$	7	C	7

Table S21. Conformers identified using NAMFIS for griselimycin in D_2O:DMSO-d_6 (4:1) solution, based on NOESY.

Conf. No.	%	Family	%
I[i] $_{\text{D2ODMSO\_full\_NOE}}$	9	A	52
II[i] $_{\text{D2ODMSO\_full\_NOE}}$	9		
VI[i] $_{\text{D2ODMSO\_full\_NOE}}$	8		
VII[i] $_{\text{D2ODMSO\_full\_NOE}}$	9		
VIII[i] $_{\text{D2ODMSO\_full\_NOE}}$	3		
XI[i] $_{\text{D2ODMSO\_full\_NOE}}$	6		
XII[i] $_{\text{D2ODMSO\_full\_NOE}}$	3		
III[i] $_{\text{D2ODMSO\_full\_NOE}}$	13	B1	33
IV[i] $_{\text{D2ODMSO\_full\_NOE}}$	9		
IX[i] $_{\text{D2ODMSO\_full\_NOE}}$	5		
X[i] $_{\text{D2ODMSO\_full\_NOE}}$	3		
XIV[i] $_{\text{D2ODMSO\_full\_NOE}}$	3		
V[i] $_{\text{D2ODMSO\_full\_NOE}}$	3	B2	3
XIII[i] $_{\text{D2ODMSO\_full\_NOE}}$	5	C	5
XV[i] $_{\text{D2ODMSO\_full\_NOE}}$	7	D	7

[i] Roman numbering indicates macrocycle conformations
[j] Family not validated

Table S22. Experimental and NAMFIS derived back-calculated interproton distances, identified using NAMFIS, for the D_2O:DMSO-d_6 (4:1) solution of griselimycin based on NOESY build-up derived data.

No	Proton A	Proton B	Experimental distance	Back-calculated distance
1	40	39	2.23	2.41
2	39	53	4.28	4.41
3	121	104	2.09	2.12
4	121	135	4.68	4.54
5	135	170-172	3.41	3.33
6	121	170-172	2.44	2.62
7	104	170-172	2.99	3.29
8	135	173-175	4.91	5.25
9	121	173-175	4.05	4.36
10	104	173-175	3.5	3.32
11	72	76	3.49	3.92
12	86	173-175	2.36	2.59
13	121	105,10	3.76	4.38
14	170-172	173-175	5.04	4.88
15	72	167-169	3.75	3.59
16	40	164-166	2.61	2.59
17	39	164-166	2.79	2.96
18	39	164-166	3.33	3.39
19	40	164-166	3.48	3.6
20	164-166	138,41-43	3.18	3.64
21	135	151	4.66	4.48
22	40	150	3.83	4.37
23	150	164-166	3.55	3.94
24	150	170-172	3.87	4.64
25	53	70	2.24	2.36
26	135	119,120	3.12	3.34
27	72	150	4.9	5.67
28	135	122	4.1	4.52
29	104	119,120	3.78	3.98
30	119,120	173-175	4.22	4.17
31	170-172	122	2.54	3
32	26	164-166	2.36	2.66
33	26	153-155	4.64	4.68
34	9	153-155	3.82	3.46
35	9	24	2.53	2.42
36	9	25	2.44	2.44
37	26	24	3.22	3.47
38	26	25	4.01	3.96
39	26	161-163	4.18	4.07
40	26	41-43,138	3.73	3.99
41	25	158-160	4.12	4.2
42	25	164-166	3.84	4.19
43	25	153-155	4.27	4.18

44	25	161-163	3.41	3.48
45	24	153-155	3.98	3.94
46	24	161-163	3.38	2.84
47	24	158-160	4.21	4.07
48	164-166	161-163	5.08	5.19
49	153-155	158-160	2.81	3.15
50	158-160	161-163	5.1	5.18
51	39	26	3.73	3.69
52	39	30	4.67	5.09
53	164-166	30	4.06	4.31

Table S23. Experimental and back-calculated (NAMFIS) scalar couplings for D_2O:DMSO-d_6 (4:1) solution of grisemlimycin

No	Proton A	Proton B	Experimental $^3J_{HH}$	Back-calculated $^3J_{HH}$
1	52	53	5.3	6.1
2	85	86	7.5	8.2
3	149	150	5.5	5.4
4	149	151	5.5	5.4
5	52	53	3.8	3.3

Table S24. The RMSD values comparing the positions of heavy atoms of the peptide backbone of griselimycin conformers, identified for D_2O:DMSO-d_6 (4:1) solution by NAMFIS. A lower RMSD value indicates higher similarity. Further distinction was made by visual comparison.

	I_A	II_A	III_B	IV_B	V_B	VI_A	VII_A	VIII_A	IX_B	X_B	XI_A	XII_A	XIII_C	XIV_B	XV_D
I_A	0														
II_A	1.72	0													
III_B	3.397	2.31	0												
IV_B	3.965	2.946	1.668	0											
V_B	3.701	2.672	1.902	1.673	0										
VI_A	1.633	1.864	2.794	3.157	2.827	0									
VII_A	1.664	2.201	3.426	3.957	3.44	1.294	0								
VIII_A	1.813	1.918	3.056	3.5	2.952	1.033	0.706	0							
IX_B	2.934	1.786	1.591	2.545	2.18	2.454	2.653	2.332	0						
X_B	3.807	2.508	2.211	2.166	1.827	3.173	3.348	2.948	1.395	0					
XI_A	2.981	1.975	2.061	1.885	2.073	2.3	2.837	2.437	1.981	1.916	0				
XII_A	1.983	1.777	2.856	3.622	3.389	2.073	1.729	1.812	1.977	2.817	2.258	0			
XIII_C	3.168	2.29	2.538	3.176	2.809	3.02	3.048	2.904	1.967	2.201	2.223	2.331	0		
XIV_B	3.098	2.302	2.039	2.538	2.628	2.923	3.356	3.163	2.388	2.814	2.193	2.825	1.651	0	
XV_D	3.221	2.433	1.7	2.179	2.063	2.885	3.357	3.082	2.014	2.526	2.295	2.922	1.978	1.412	0

6.3. Conformers identified for the D_2O/DMSO-d_6 solution using NAMFIS, based on ROESY data

Table S25. Conformers identified using NAMFIS for the macrocyclic core of griselimycin in D_2O:DMSO-d_6 (4:1) solution, based on ROESY build-ups

Conf. No.[k]	Population (%)	Conformational family	Population (%)
I[k] D2ODMSO_MC_ROE	20	A1	35
II[k] D2ODMSO_MC_ROE	3		
III[k] D2ODMSO_MC_ROE	12		
IV[k] D2ODMSO_MC_ROE	26	A2	26
V[k] D2ODMSO_MC_ROE	31	B	31
VI[k] D2ODMSO_MC_ROE	3	C	7
VII[k] D2ODMSO_MC_ROE	4		

Table S26. Conformers identified using NAMFIS for griselimycin in D_2O:DMSO-d_6 (4:1) solution, based on ROESY build-ups

Conf. No.[l]	Population (%)	Conformational family	Population (%)
**16[l] D2ODMSO_full_ROE**	26	A1	26
**13[l] D2ODMSO_full_ROE**	19	A2	24
**14[l] D2ODMSO_full_ROE**	5		
**8[l] D2ODMSO_full_ROE**	14	B1	24
**9[l] D2ODMSO_full_ROE**	4		
**10[l] D2ODMSO_full_ROE**	6		
**11[l] D2ODMSO_full_ROE**	14	B2	14
**12[l] D2ODMSO_full_ROE**	8	C1	8
**15[l] D2ODMSO_full_ROE**	3	C2	3

Table S27. Experimental and NAMFIS derived back-calculated interproton distances, identified using NAMFIS, for the D_2O:DMSO-d_6 (4:1) solution of griselimycin based on ROESY build-up derived data

No	Proton A	Proton B	Experimental distance	Back calculated distance
1	40	39	2.27	2.38
2	121	104	2.08	2.13
3	135	170-172	3.97	3.85
4	121	170-172	2.79	2.68

[k] Roman numbering indicates macrocycle conformations
[l] Arabic numbers indicate griselimycin conformations

5	104	173-175	3.63	3.36
6	40	138,41-43	2.61	2.59
7	39	138,41-43	2.97	2.97
8	39	164-166	3.69	3.63
9	40	164-166	4.38	4.33
10	164-166	138,41-43	3.29	3.54
11	53,151	70,25	2.89	2.86
12	104	119,120	4.4	4
13	53,151	71	2.89	2.83
14	150	138,41-43	4.33	4.6
15	150	164-166	3.89	4.01
16	150	170-172	3.87	4.33
17	71	167-169	3.78	3.64
18	173-175	105,10	2.92	2.98
19	26	164-166	2.4	2.5
20	9	70,25	2.75	2.75
21	9	24	2.39	2.39
22	26	70,25	3.26	3.75
23	26	138,41-43	4.66	4.71
24	39	26	4.99	4.56
25	24	30,122,123	2.63	2.71
26	70,25	161-163	3.75	3.83
27	70,25	167-169	3	2.87
28	30,122,123	161-163	2.84	2.63

Table S28. Experimental and back-calculated (NAMFIS) scalar couplings for D2O:DMSO-*d*$_6$ (4:1) solution of griselimycin

No	Proton A	Proton B	Experimental $^3J_{HH}$	Back-calculated $^3J_{HH}$
1	52	53	5.3	5.4
2	85	86	7.5	8
3	149	150	5.5	5.6
4	149	151	5.5	6
5	52	53	3.8	3.6

Table S29. The RMSD values comparing the positions of heavy atoms of the peptide backbone of griselimycin conformers, identified for D_2O:DMSO-d_6 (4:1) solution by NAMFIS using ROESY build-up data. A lower RMSD value indicates higher similarity. Further distinction was made by visual comparison.

	8_B1	9_B1	10_B1	11_B2	12_C1	13_A2	14_A2	15_C2	16_A1
8_B1	0								
9_B1	1.017	0							
10_B1	0.903	0.558	0						
11_B2	1.474	1.556	1.761	0					
12_C1	1.954	1.825	1.883	2.214	0				
13_A2	3.232	2.722	2.565	3.68	3.286	0			
14_A2	3.343	2.906	2.689	3.818	3.408	0.529	0		
15_C2	2.312	2.158	1.937	2.942	2.101	2.691	2.725	0	
16_A1	4.434	3.875	3.785	4.652	4.163	2.015	2.034	3.524	0

Table S30. Populated conformers of griselimycin in D_2O:DMSO-d_6 (4:1) solution (based on ROESY). Graphical representation including intramolecular hydrogen bonds. Conformers (pdb) are provided on Zenodo via https://doi.org/10.5281/zenodo.19458261

No	Conf	Family	[%]
16<br>**D2ODMSO_full_ROE**	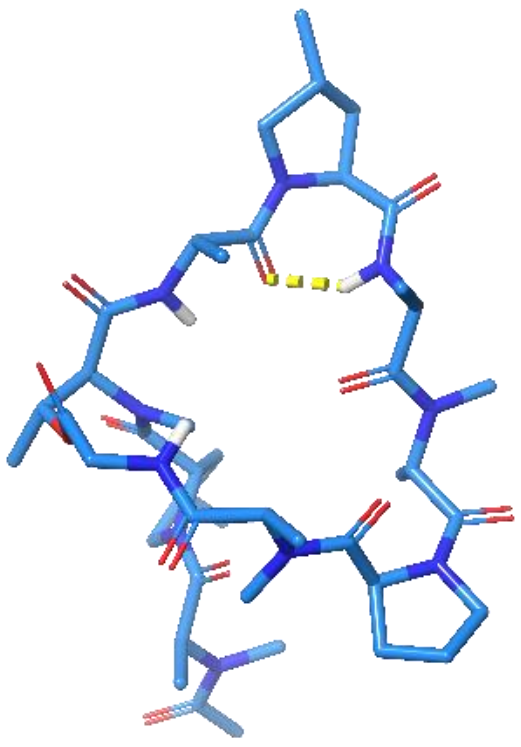	A1	26
13<br>**D2ODMSO_full_ROE**	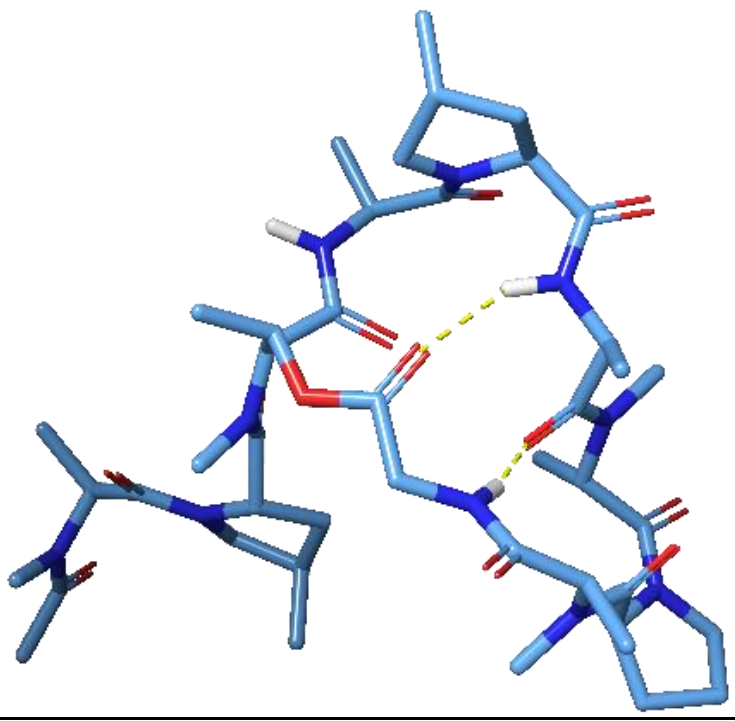	A2	19

14<br>**D2ODMSO_full_ROE**	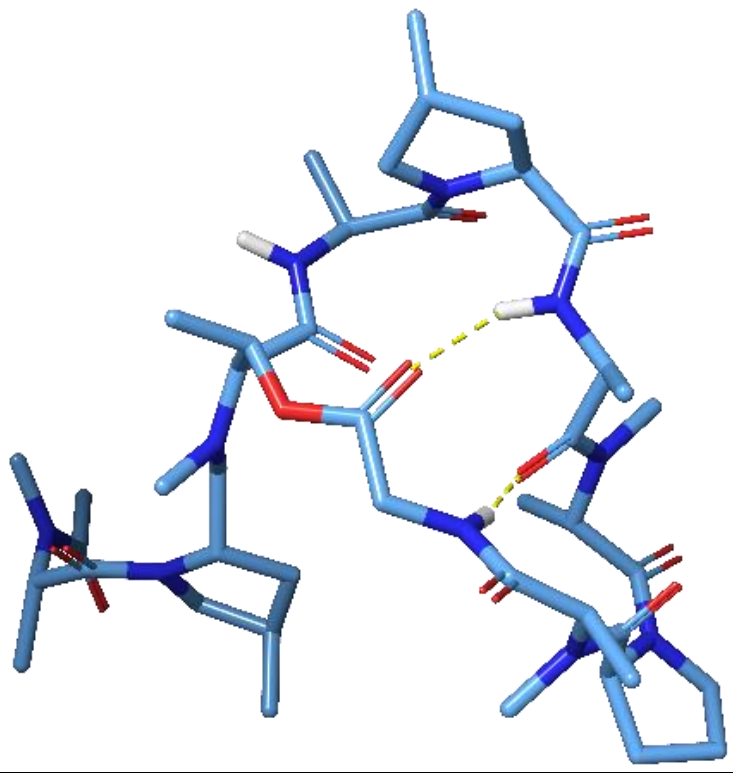	A2	5
8<br>**D2ODMSO_full_ROE**	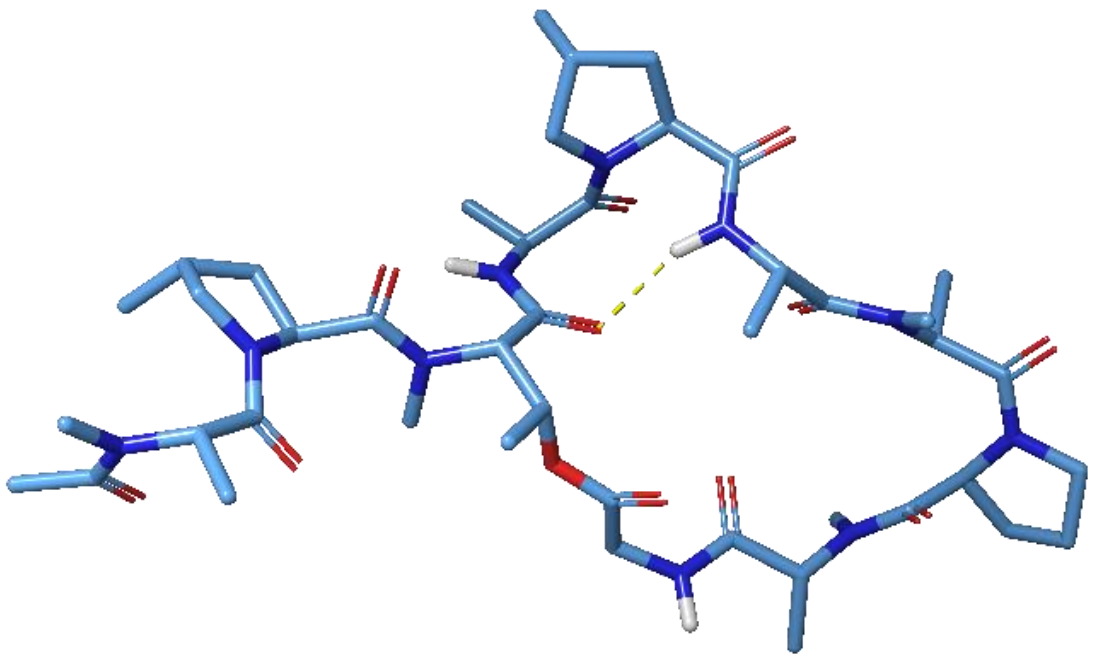	B1	14
10<br>**D2ODMSO_full_ROE**	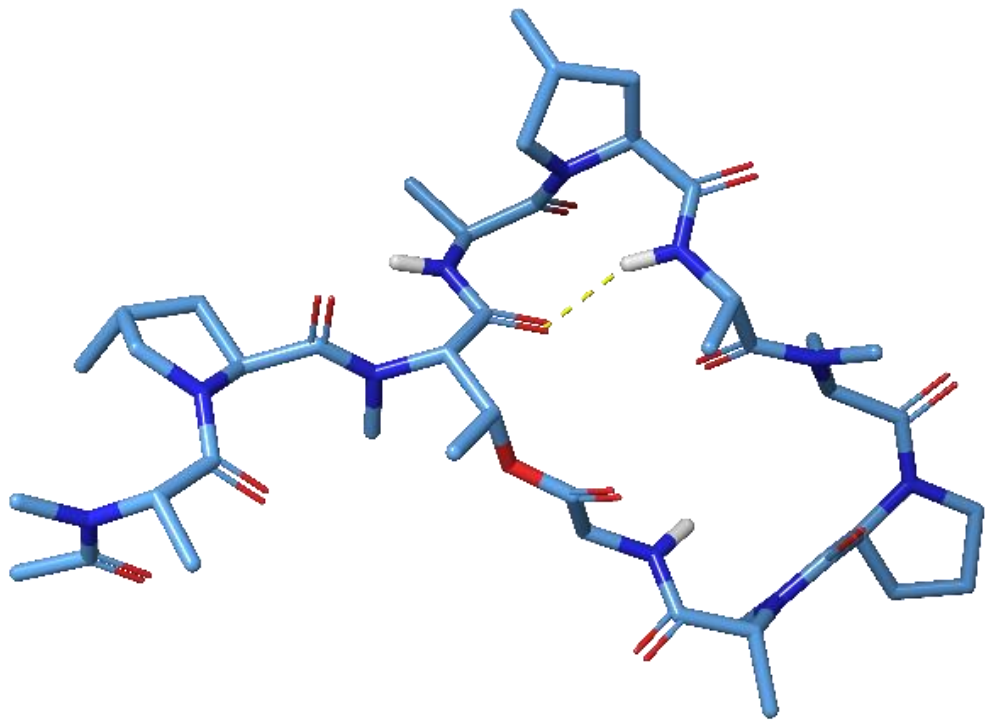	B1	6
9<br>**D2ODMSO_full_ROE**	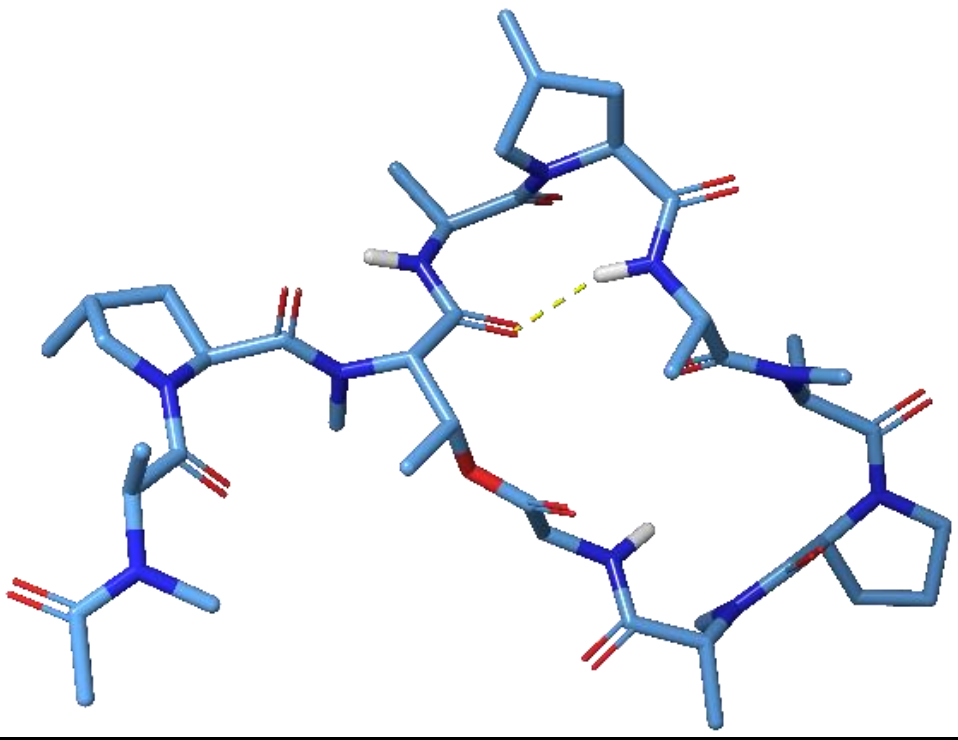	B1	4
11<br>**D2ODMSO_full_ROE**	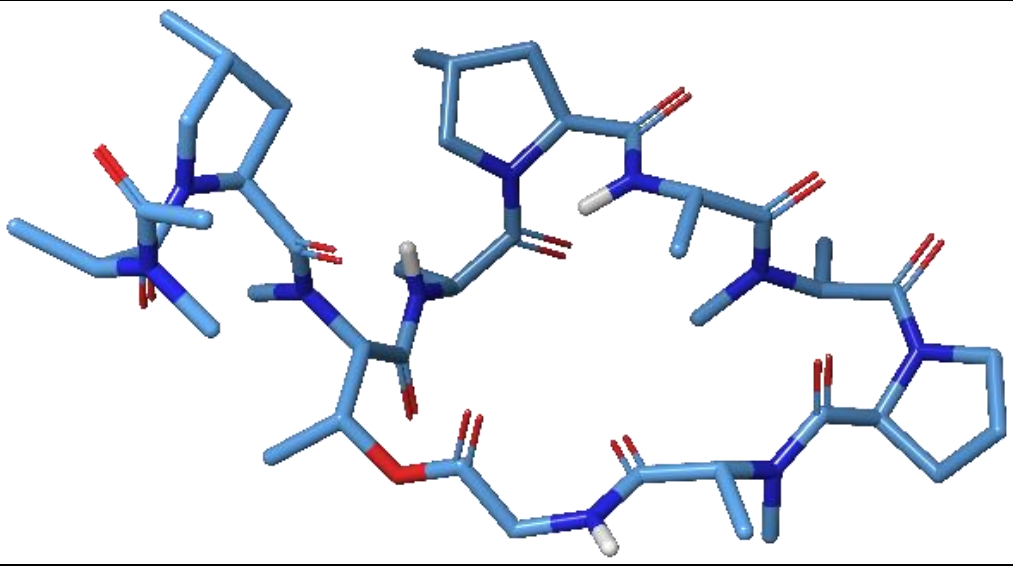	B2	14

12<br>**D2ODMSO_full_ROE**	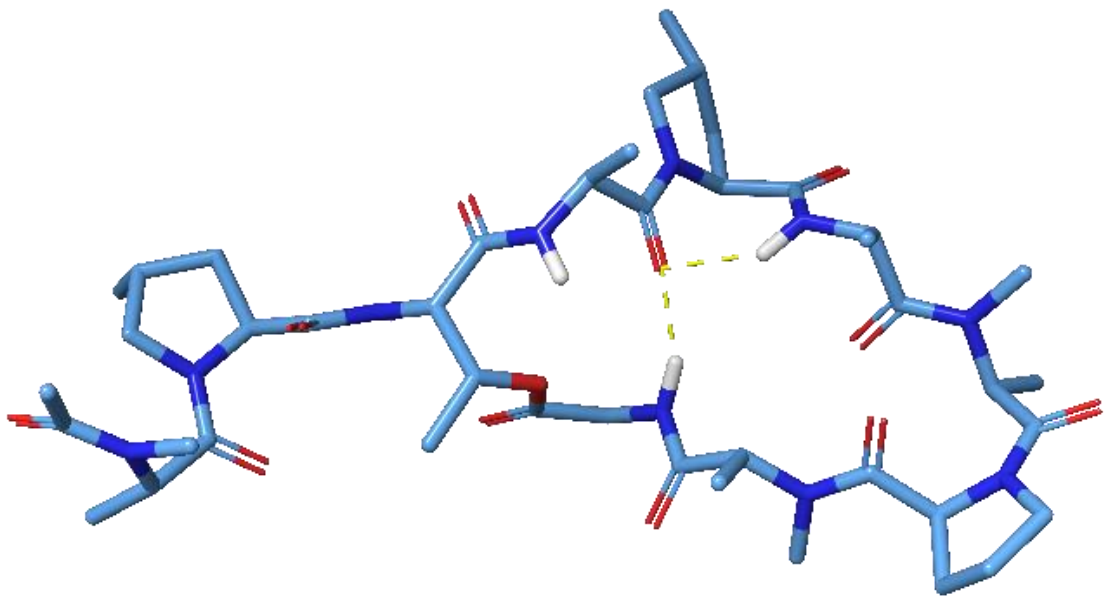	C1	8
15<br>**D2ODMSO_full_ROE**	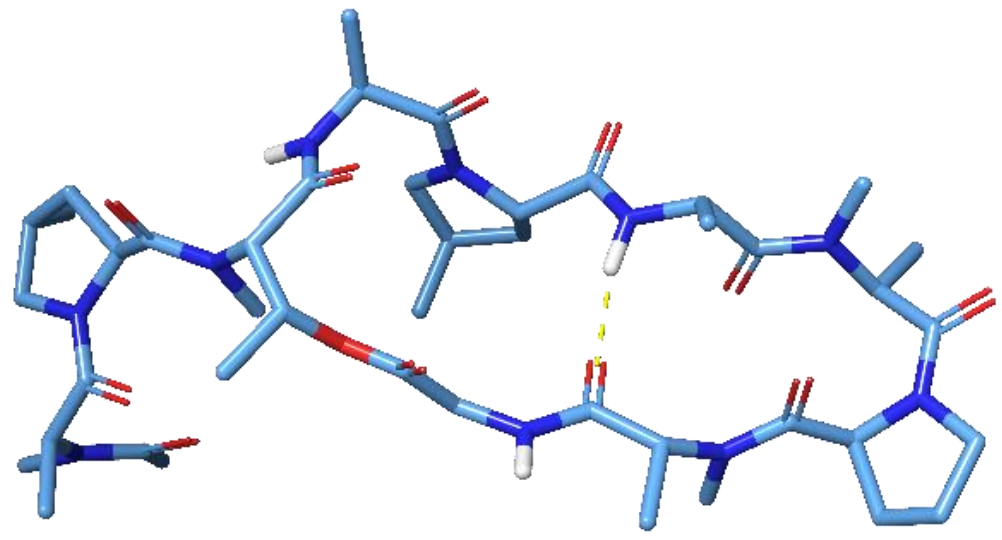	C2	3

7. Molecular descriptors

7.1 Calculated descriptors for conformational ensemble in $CDCl_3$ solution[3]

Table S31. Calculated molecular descriptors for griselimycin in $CDCl_3$: Radius of gyration (R_{gyr}), total solvent accessible surface area (Total 3D SA), solvent accessible polar surface area (SA 3D PSA) and solvent accessible non-polar surface area (SA 3D NPSA)

Conformer	[%]	R_{gyr} [Å]	Total 3D SA [Å^2]	SA 3D PSA [Å^2]	SA 3D NPSA[g] [Å^2]
1$_{\text{CDCl3_full}}$	17	6.75	1367.02	123.68	1243.34
2$_{\text{CDCl3_full}}$	8	6.49	1360.79	136.91	1223.88
3$_{\text{CDCl3_full}}$	4	5.98	1344.05	154.97	1189.08
4$_{\text{CDCl3_full}}$	9	6.3	1398.35	175.99	1222.36
5$_{\text{CDCl3_full}}$	13	6.57	1361.94	141.29	1220.65
6$_{\text{CDCl3_full}}$	8	6.98	1395.48	130.87	1264.61
7$_{\text{CDCl3_full}}$	41	6.56	1417.96	165.22	1252.74

7.2 Calculated descriptors for ensemble in D_2O:DMSO-d_6 (4:1) solution, based on ROESY data[3]

Table S32. Calculated molecular descriptors for griselimycin in D_2O:DMSO-d_6 (4:1) - (ROESY only): Radius of gyration (R_{gyr}), total solvent accessible surface area (Total 3D SA), solvent accessible polar surface area (SA 3D PSA) and, solvent accessible non-polar surface area (SA 3D NPSA)

Conformer	[%]	R_{gyr} [Å]	Total 3D SA [Å^2]	SA 3D PSA [Å^2]	SA 3D NPSA[m][Å^2]
8$_{\text{D2ODMSO_full_ROE}}$	14	6.63	1366.34	150.99	1215.35

[m] Calculated by subtracting SA 3D PSA from Total 3D SA

9 D2ODMSO_full_ROE	4	6.44	1345.93	156.63	1189.3
10 D2ODMSO_full_ROE	6	6.5	1345.86	151.98	1193.88
11 D2ODMSO_full_ROE	14	6.13	1283.99	127.32	1156.67
12 D2ODMSO_full_ROE	8	6.62	1345.88	134.94	1210.94
13 D2ODMSO_full_ROE	19	5.85	1283.72	125.25	1158.47
14 D2ODMSO_full_ROE	5	5.88	1306.54	129.88	1176.65
15 D2ODMSO_full_ROE	3	6.61	1350.71	121.65	1229.06
16 D2ODMSO_full_ROE	26	5.47	1196.16	157.34	1038.82

7.3 Experimental polar surface area (EPSA)

Table S33. EPSA observed for griselimycin. Supercritical fluid chromatography (SFC) on Phenomenex Chirex3014 (S)Val and (R)NEA 250 x 4.6 mm; Flowrate: 5 mL/min; Column temperature: 40 °C; back pressure: 140 bar.

(A): Supercritical CO_2

(B): Ammonium formate 20 mM in HPLC grade Methanol

Gradient:

t = 0 min: 95% (A), 5% (B)

t = 11min: 40% (A), 60% (B)

t = 16 min: 40% (A), 60% (B)

t = 17 min: 95% (A), 5% (B)

SFC retention time [min]	3.98
Mass [g/mol]	1112.7
Detection wavelength [nm]	215
EPSA [$Å^2$]	74

8. References

(1) Schmieder, P. and Kessler, H., Determination of the ϕ angle in a peptide backbone by NMR spectroscopy with a combination of homonuclear and heteronuclear coupling constants. *Biopolymers* **1992**, 32 (4), 435-440. https://doi.org/10.1002/bip.360320421.

(2) Pérez, C.; Löhr, F.; Rüterjans, H.; Schmidt, J. M. Self-consistent Karplus parametrization of ^3J couplings depending on the polypeptide side-chain torsion X1. *J. Am. Chem. Soc.* **2001**, *123* (29), 7081–7093. https://doi.org/10.1021/ja003724j.

(3) Pedretti A, Villa L, Vistoli G. VEGA: a versatile program to convert, handle and visualize molecular structure on Windows-based PCs. *J. Mol. Graph. Model.* **2002**, 21(1), 47-9. doi: 10.1016/s1093-3263(02)00123-7. http://www.vrml.org/fs.
